# Activation of lysosomal mediated cell death in the course of autophagy by mTORC1 inhibitor

**DOI:** 10.1038/s41598-022-07955-1

**Published:** 2022-03-23

**Authors:** Sameer Ullah Khan, Anup Singh Pathania, Abubakar Wani, Kaneez Fatima, Mubashir Javed Mintoo, Baseerat Hamza, Masroor Ahmad Paddar, Wadhwa Bhumika, Loveleena Kour Anand, Mir Shahid Maqbool, Sameer Ahmad Mir, Jaspreet Kour, Vunnam Venkateswarlu, Dilip Manikrao Mondhe, Sanghapal D. Sawant, Fayaz Malik

**Affiliations:** 1 Pharmacology Division, CSIR-Indian Institute of Integrative Medicine, Sanat Nagar, Srinagar, Jammu and Kashmir 190005 India; 2 Academy of Scientific and Innovative Research (AcSIR), Ghaziabad, Uttar Pradesh, 201002 India; 3 Medicinal Chemistry, CSIR-Indian Institute of Integrative Medicine, Jammu, India

**Keywords:** Biochemistry, Biotechnology, Cancer, Drug discovery, Neuroscience, Stem cells, Pathogenesis

## Abstract

Lysosomal biogenesis plays a vital role in cell fate. Under certain conditions, excessive lysosomal biogenesis leads to susceptibility for lysosomal membrane permeabilization resulting in various pathological conditions including cell death. In cancer cells apoptosis machinery becomes dysregulated during the course of treatment, thus allows cancer cells to escape apoptosis. So it is therefore imperative to identify cytotoxic agents that exploit non-apoptotic mechanisms of cell death. Our study showed that pancreatic cancer cells treated with SDS-203 triggered an incomplete autophagic response and a nuclear translocation of transcriptional factor TFEB. This resulted in abundant biosynthesis and accumulation of autophagosomes and lysosomes into the cells leading to their death. It was observed that the silencing of autophagy genes didn’t alter the cell fate, whereas siRNA-mediated silencing of TFEB subdued SDS-203 mediated lysosomal biogenesis and associated cell death. Further mouse tumors treated with SDS-203 showed a significant reduction in tumor burden and increased expression of lysosomal markers. Taken together this study demonstrates that SDS-203 treatment triggers non-apoptotic cell death in pancreatic cancer cells through a mechanism of lysosome over accumulation.

## Introduction

Autophagy is the conserved catabolic cellular process in which lysosomal enzymes degrade defective or excess organelles and unwanted proteins^1^. It is also important for the recycling of metabolites for cell survival, thereby playing a definite role in maintaining cellular homeostasis^2,3^. Autophagy is activated under various stress conditions such as amino acid starvation, unfolded protein response and is inhibited by the high energy state of the cell through activation of mammalian target of rapamycin (mTORC1)^4,5^. mTORC1 is a serine/threonine kinase that regulates cellular metabolism and promotes cell growth^6,7^. mTORC1 activation inhibits mammalian autophagy directly by inhibiting ULK1 complex formation and at transcriptional level it regulates autophagy by modulating the subcellular localization of transcription factor EB (TFEB)^8,9,10^. The deregulation of autophagy pathway compromises cell survival and its association has been found to play a role in many diseases like cancer^11^. Autophagy-lysosome pathway is associated with various cancer hallmarks like death resistance, escaping immune surveillance and deregulation of metabolism^12^. It is established that some invasive cancers s like pancreatic adenocarcinoma are resistant to Type I (apoptotic) and Type II (autophagic) cell death^13-17^; wherein the activation of nonclassical death pathways through small molecules offers alternate means to address these challenges. Pancreatic cancer is one of the most aggressive human malignancies and is the fourth major cause of cancer-related deaths^18,19^. Generally, the survival rate of patients with pancreatic adenocarcinoma is very low because of the late diagnosis and poor response to available treatments^20^. Moreover, pancreatic tumor dormancy, relapse along with chemo-resistance liked with autophagy is a major concern for the current available therapies^21-24^. Studies have shown that some anti-cancerous agents trigger protective autophagy in pancreatic cancer cells and rescue them from apoptosis^25^. Therefore, identification of alternate target-based therapies to circumvent this challenge is a huge unmet medical need. In this study, we identified a novel mTORC1 inhibitor SDS-203 causing non-apoptotic cells death in aggressive MIA PaCa-2 cells. It was observed that SDS-203 triggered early autophagy with simultaneous inhibition of autophagosomes-lysosome fusion, leading to excessive lysosomal accumulation and cancer cell death. SDS-203 treatment in tumor bearing mice showed significant reduction of tumor burden, an effect that was diminished by using lysosomal quencher NH4Cl. The present finding is of significant clinical importance and brings a me chanistic rationale for the prospective use of SDS-203 in the treatment of pancreatic cancer.

## Results

### SDS-203 induced no-apoptotic death in pancreatic cancer cells

**Figure 1:**
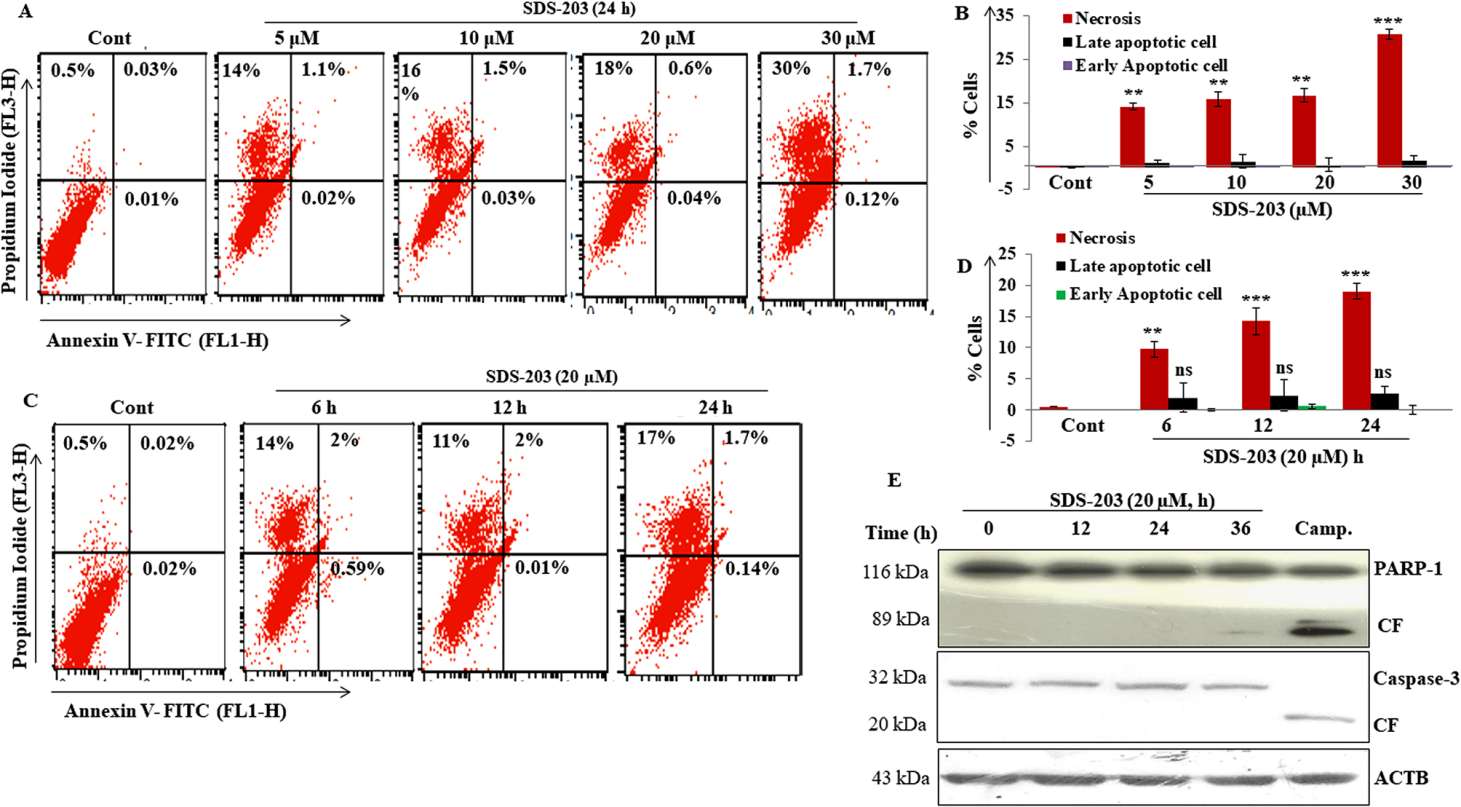
SDS-203 decreases cell viability by the non-apoptotic way in pancreatic cancer cell line MIA PaCa-2. (**A**–**D**) MIA PaCa-2 cells were incubated with different concentrations of SDS-203 (5, 10, 20, 30 µM) for 24 h or single concentration (20 µM) for various time points (0, 6, 12, 24 h) then cells were stained with Annexine-V-FITC and PI (1 µg/mL) followed by quantification of apoptotic cells by flowcytometry (**E**) MIA PaCa-2 cells were treated with SDS-203 (20 μM) in a time-dependent manner (0, 12, 24, 36 h) and protein expression of cleaved PARP1 and cleaved Caspase3 were determined by western blotting, camptothecin (1 µM) was taken as a positive control. Each experiment was repeated three times. *p*-value represents: **p < 0.01; ***p < 0.001vs. control.

In our earlier report, we showed that SDS-203 targets mTORC1 and inhibits the cell growth of MIA PaCa-2 cells with IC50 7 ± 1.6 µM^26^. In our current studies, we further tried to understand the mechanism of SDS-203 mediated cell death in MIA PaCA-2 cells, which happened to be non-apoptotic in nature. The initial attempts to characterize the nature of SDS-203 induced cell death via apoptotic pathway were analyzed by flowcytometry and western blotting. Flowcytomtric analysis of Annexin V and propidium iodide (PI) stained MIA PaCa-2 cells revealed that SDS-203 caused non-apoptotic cell death and a large number of treated cells were found in PI^+^ quadrant depicting necrosis (Fig. 1A–D). This was further confirmed as SDS-203 treatment failed to induce PARP1 or caspase 3 cleavage (Fig. 1E). Free radical generation like reactive oxygen species (ROS) was observed through flowcytometry by using DCFDA that remained unchanged during the course of SDS-203 treatment in MIA PaCa-2 cells (Supplementary Fig. 1a, b).

### SDS-203 treatment induced initial autophagic response in MIA PaCa-2 cells

**Figure 2:**
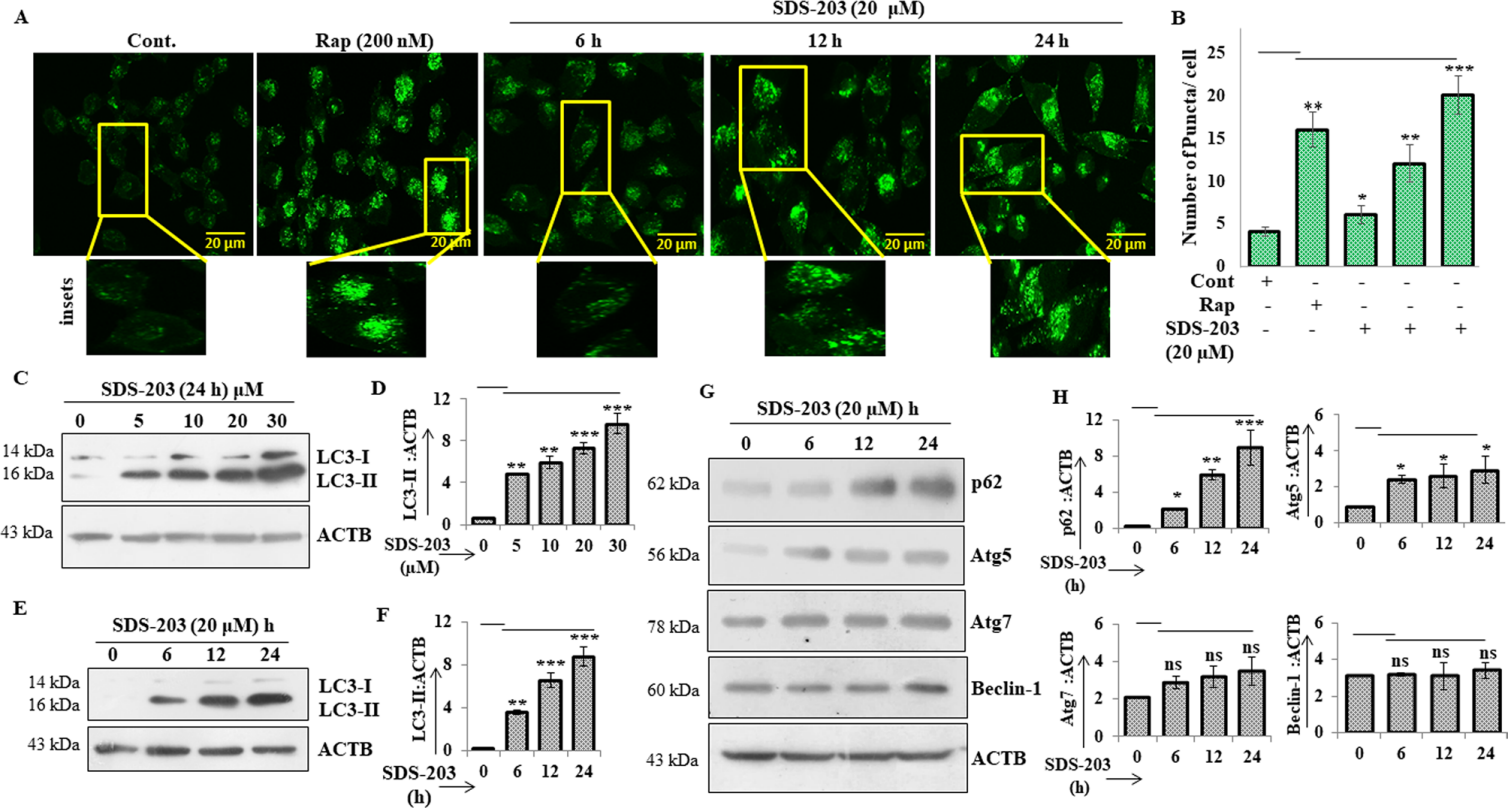
SDS-203 initiates autophagy in l MIA PaCa-2 cells. (**A**, **B**) Representative fluorescent images of transiently transfected (GFP-LC3) pancreatic cancer cells treated with SDS-203 (20 µM) for 6, 12, 24 h and rapamycin (200 nM) as a positive control. Quantification of 30 random cells were taken for each experiment. (**C**–**F**) Represents protein expression levels of LC3-II in MIA PaCa-2 cells when treated with various concentrations (0, 5, 10, 20, 30 μM; 24 h) or different time points (0, 6, 12, 24 h; 20 µM) of SDS-203. (**G**, **H**) MIA PaCa-2 cells were subjected to SDS-203 (20 μM) in a time-dependent manner and expression of p62, ATG5, ATG7, Beclin-1 were analyzed by western blotting. Densitometry quantification of protein expression was done by using ImageJ software. All data are presented as the mean of SD from 3 independent experiments. Statistics, Student’s *t* test *p < 0.05, **p < 0.01, ***p<0.001 vs. control (0.1%DMSO).

To observe the effect of SDS-203 treatment on the autophagic response in MIA PaCa-2, various experiments were performed. SDS-203 treated cells were stained with fluorescent Acridine Orange (AO) dye (1 μg/mL), and cells were observed under fluorescence microscopy. It was found that SDS-203 significantly increased AO acid vesicles in MIA PaCa-2 cells compared to that of control (Supplementary Fig. 2a, b). Further, we transiently transfected MIA PaCa-2 cells with fluorescent autophagosomal markers GFP-LC3 and followed by treatment with SDS-203 (20 μM). Microscopic analysis clearly showed that SDS-203 increased GFP-LC3 punctation by five fold, when compared to control (Fig. 2A, B). Treatment of SDS-203 upregulates the conversion of LC3-1 to LC3-II as shown by western blotting (Fig. 2C–D) reflecting the initiation of autophagy process. Expression of other important autophagic proteins ATG5, ATG7 were slightly affected, while Bec lin-1 expressi on remained unchanged by SDS-203 treatment as shown in Figure 2G, H. However, the expression of an adapter protein p62/SQSTM1 (Sequestosome-1) was surprisingly upregulated by several folds, indicating the blockage in autophagy flux. Thus indicating that SDS-203 treatment triggers the initiat ion of defective auto phagic response in MIA PaCa-2 cells.

### SDS-203 triggers initiation of autophagy by mTORC1 inhibition

**Figure 3:**
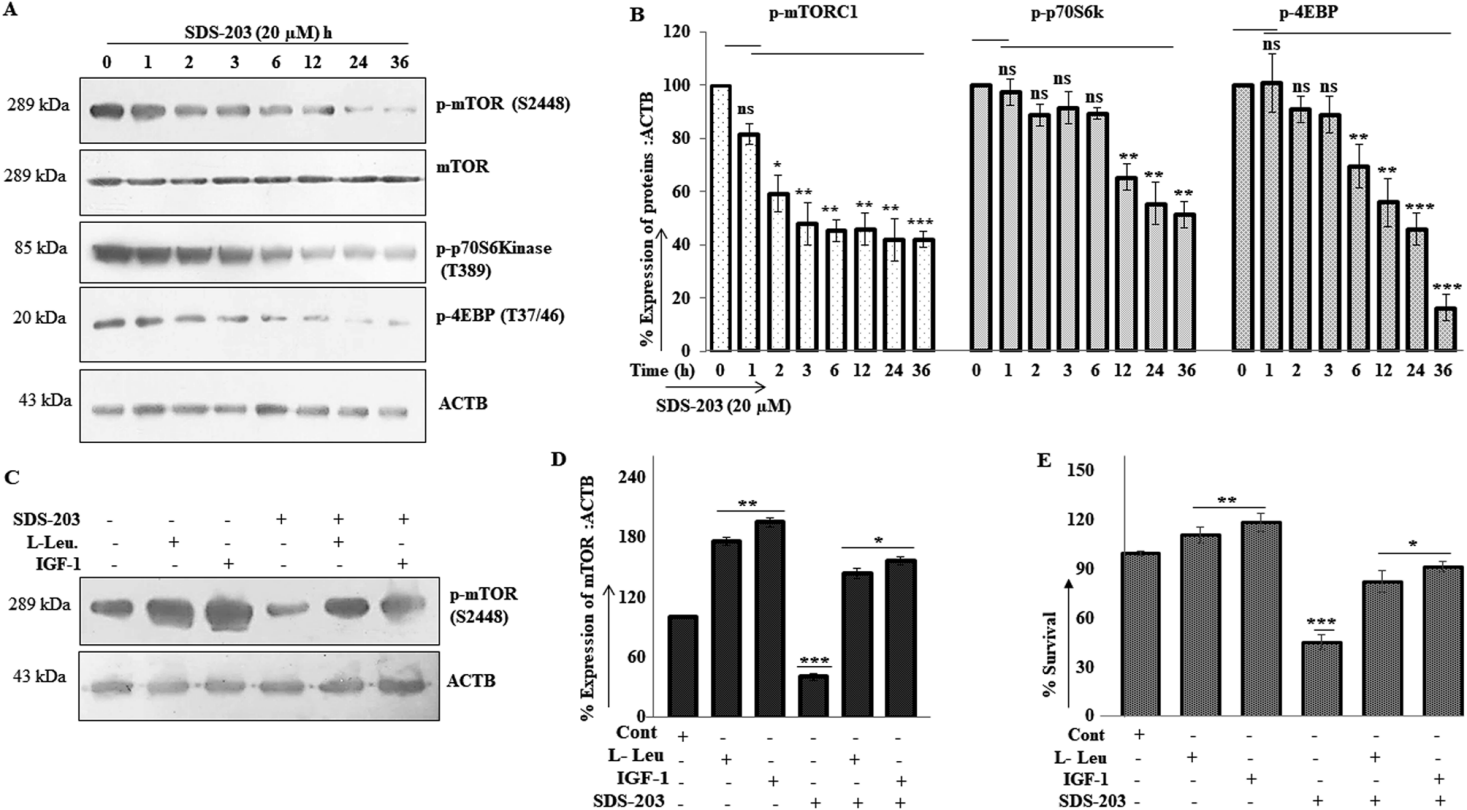
SDS-203 induces autophagy by inhibiting mTORC1 activity. (**A**, **B**) MIA PaCa-2 cells were treated with SDS-203 (20 μM) for various time points (0, 1, 2, 3, 6, 12, 24, 36 h) and its effect on p-mTORC1 along with its downstream target s– p-p70S6kinase and p-4EBP1, were analyzed and quantified. (**C**, **D**) MIA PaCa-2 cells were incubated with SDS-203 (20 µM) for 24 h in the presence or absence of IGF-1 (50 ng) or l-leucine (0.5 mM). Protein expression was analyzed and quantified by using ImageJ software. (**E**) Graphical representation (MTT) of survival percentage of MIA PaCa-2 cells treated with SDS-203 (20 µM) for 48 h in presence or absence of l-leucine (0.5 mM) or IGF-1 (50 ng). All data are presented as the mean of SD from three independent experiments. Statistics, Student’s *t* test *p < 0.05, **p < 0.01, ***p<0.001 vs. control (0.1% DMSO).

Our results confirmed that treatment of SDS-203 (20 µM) at various time points inhibited mTORC1 and its downstream substrates activity significantly (Fig. 3A, B). mTORC1 is a nutrient-sensing kinase that regulates autophagy in cells^27^. Further confirmation showed that SDS-203 action was diminished in presence of known mTORC1 activators—insulin-like growth factor1 (IGF-1)^28^ and l -leucine^29^ when compared to SDS-203 only treated cells, shown in Figure 3C, D. Cell viability assay (MTT, 48 h) of the same samples demonstrates that IGF or l-leucine treated cells rescued cells death in SDS-203 treated cells (Fig. 3E). These results clearly indicate that SDS-203 mediated inhibition of mTORC1 causing initiation of autophagy.

### SDS-203 inhibits autophagy flux in MIA PaCa-2 cells

**Figure 4:**
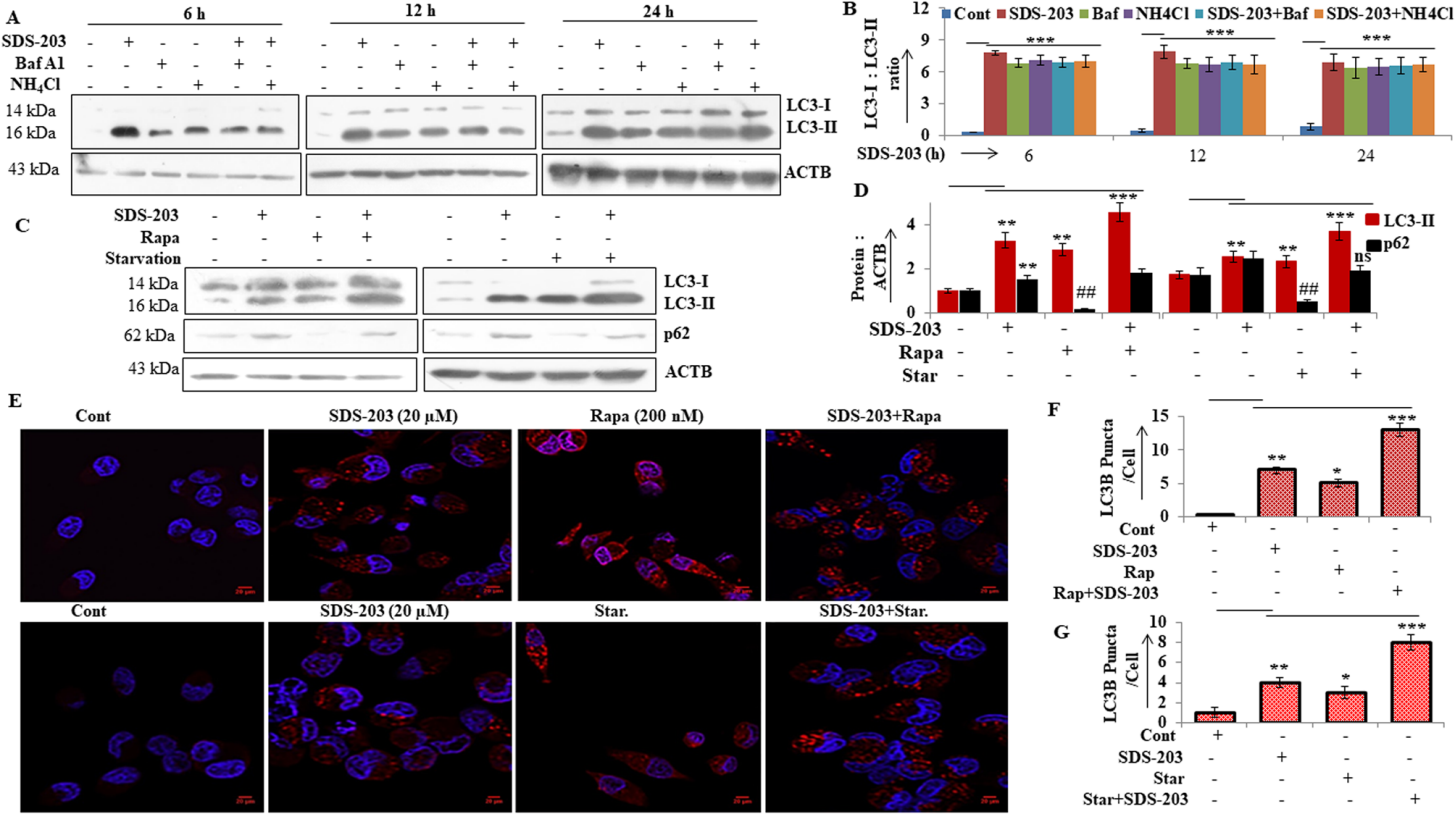
SDS-203 initiates incomplete autophagy by inhibiting autophagy flux. (**A**, **B**) MIA PaCa-2 cells were treated with SDS-203 (20 μM) for 6, 12 and 24 h in presence or absence of bafilomycin A1 (80 nM) or NH_4_Cl (10 mM) and LC3 turnover was detected and quantified by western blot analysis. (**C**, **D**) SDS-203 (20 µM) treated MIA PaCa-2 cells were allowed to grow in the presence or absence of rapamycin (200 nM, 12 h) or starved for (12 h), followed by protein expression examination of LC3-II and p62. (**E**–**G**) MIA PaCa-2 cells were incubated with SDS-203 in the presence or absence of rapamycin or starvation and the effects on LC3-II expression was visualized using confocal microscopy, LC3-II puncta quantification was done by using ImageJ software; the panel represents the set of images of at least three independent biological replicates. Statistics, Student’s *t* test *p < 0.05, **or##*p* < 0.01, ***p < 0.001 vs. control (0.1% DMSO).

As revealed in Figure 2G the possibility of autophagy flux blockade or fusion defects between autophagosome and lysosomes in MIA PaCa-2 cells upon SDS-203 treatment. We treated cells with SDS-203 in the presence or absence of autophagy inhibitors—bafilomycin A1 or NH_4_Cl. Western blotting analysis indicated that SDS-203 failed to further upregulate LC3-II expression in pancreatic cancer cells treated with bafilomycin A1 or NH_4_Cl when compared to SDS-203 only treated cells for 6, 12 and 24 h (Fig. 4A, B). On the contrary LC3-II and p62 expression was upregulated upon co-treatment of cells with SDS-203 and autophagy activator rapamycin or starvation when compared with control or individual treatments (Fig. 4C, D). Microscopic examination for endogenous LC3-II puncta formation was intensified in the combined presence of SDS-203 and autophagy inducers when compared with individual treatments (Fig. 4E–G). These results demonstrated that SDS-203 impairs autophagy flux which leads to the accumulation of autophagosomes and lysosomes thus acts as a potent inhibitor of autophagy maturation.

### SDS-203 induces lysosomal biogenesis and accumulation in pancreatic cancer cells

**Figure 5:**
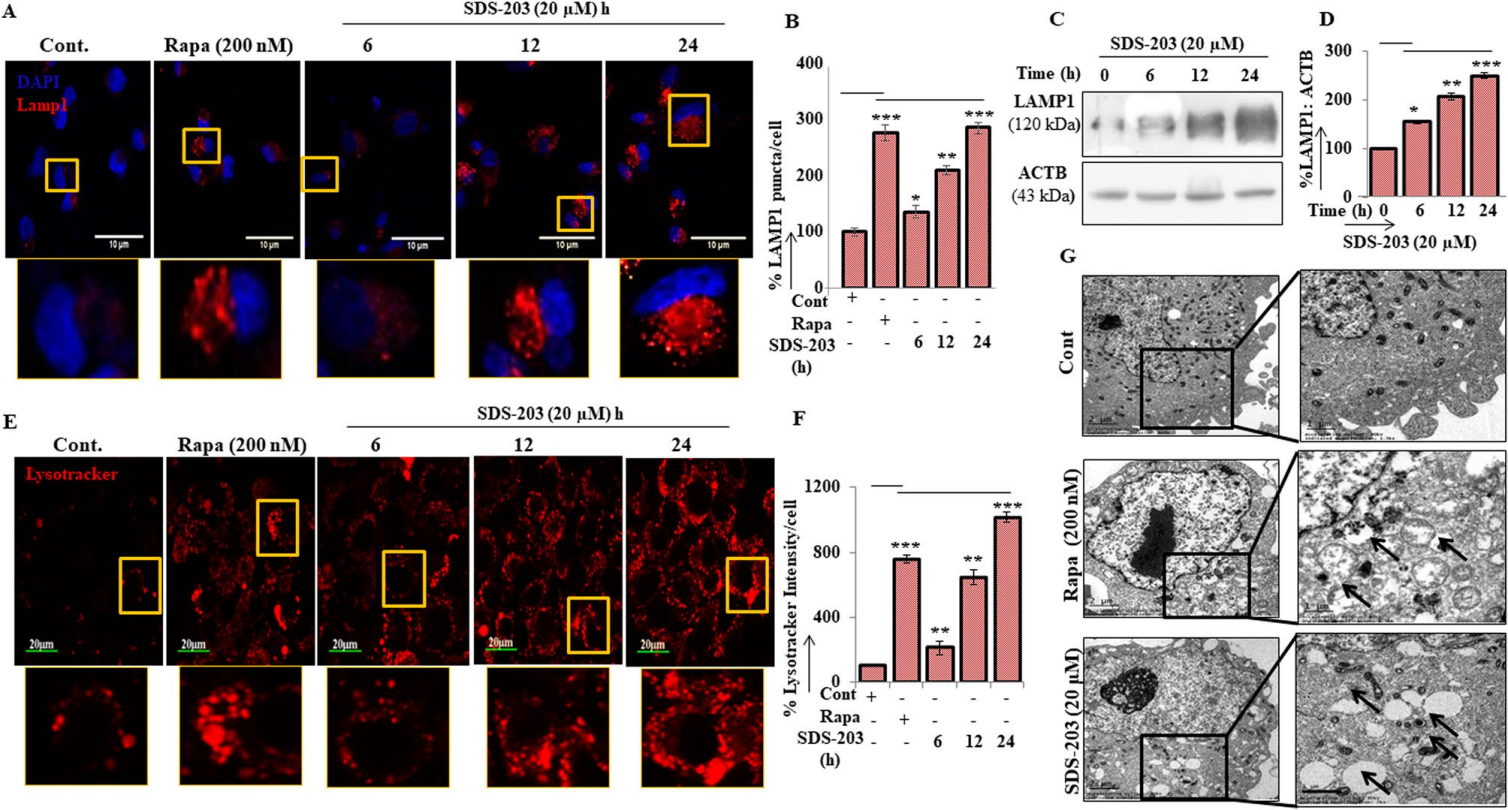
SDS-203 induces lysosomal biogenesis in MIA PaCa-2 cells. (**A**, **B**) Represent confocal images and puncta density of LAMP1expression upon treatment of SDS-203 (20 µM) for 6, 12, 24 h or rapamycin (200 nM) for 12 h. (**C**, **D**) Demonstrates LAMP1 protein expression (Western botting) and quantification in MIA PaCa-2 cells incubated with SDS-203 (20 µM) for different time points. (**E**, **F**) Demonstrate respective lysotracker DND-99 probe (50 nM) staining and quantification of fluorescent signal in MIA PaCa-2 cell treated with SDS-203 (20 µM) for different time points. (**G**) Represents the accumulation of unfused autophagosomes and lysosomes in pancreatic cancer cells upon SDS-203 treatment versus autolysosomes formation in rapamycin-treated samples, visualized under electron microscopy. Statistics, Student’s *t* test *p < 0.05, **p < 0.01, ***p < 0.001 vs. control (0.1%DMSO).

In the present study, we investigated the lysosomal status in the course of SDS-203 treatment in MIA PaCa-2 cells. Immunofluorescence and immunoblot analysis of lysosomes marker protein LAMP1 revealed that SDS-203 treatment significantly upregulates LAMP-1 expression in MIA PaCa-2 cells (Fig. 5A–D). In addition, SDS-203 treatment upregulated the quantity of the lysosomes observed by confocal microscopy (Fig. 5E, F) and flowcytometry (Supplementary Fig. 3a, b) using lysotracker staining. Further evaluation by electron microscopy clearly established that SDS-203 resulted in upregulation and accumulation of lysosomes and autophagosome in MIA PaCa-2 cells (Fig. 5G).

### SDS-203 suppresses autophagy at late stage without affecting lysosomal function and is mediated through suppression of mTORC1

**Figure 6:**
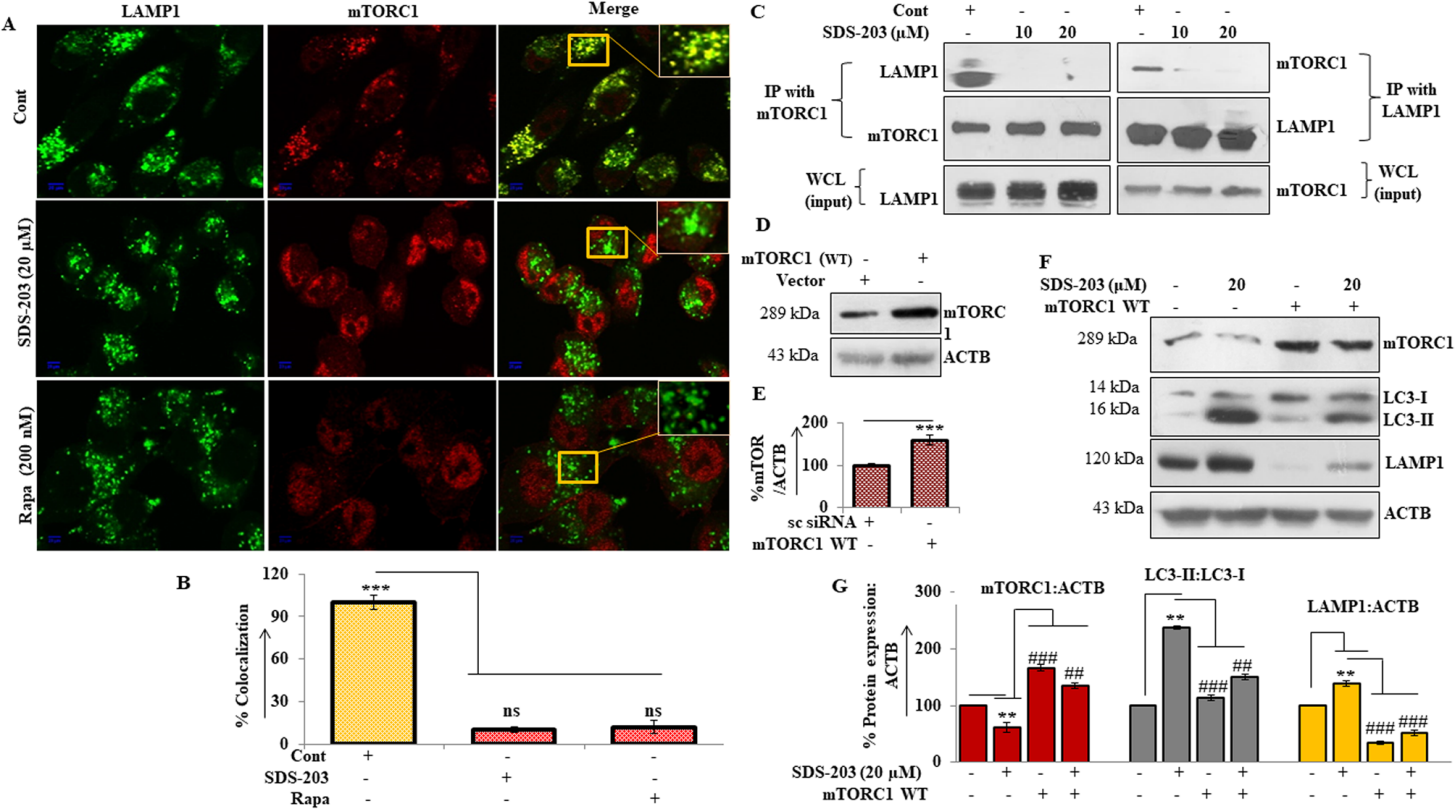
SDS-203 participates in lysosomes and autophagosome fusion inhibitor. (**A**) MIA PaCa-2 cells treated with SDS-203 prior to fluorescent antibody labeling against endogenous LAMP1 (green) and mTORC1 (red) colocalization (yellow). (**B**) Shows treatment colocalization correlation compared to control. (**C**) Pancreatic cancer cells were treated with SDS-203 (0, 10, 20 µM) and taken for mTORC1 and LAMP1 interaction investigation by immunoprecipitation (*ip* immune precipitation, *WCL* whole cell lysate). (**D**, **E**) Represents the expression of mTORC1 in vector or WT mTORC1 plasmid transfected cells and its quantification respectively. (**F**) SDS-203 treated to mTORC1 overexpressed MIA PaCa-2 cells and expression of LC3-II and LAMP1 were detected. (**G**) Densitometry of blots. Each experiment was repeated thrice, ip experimentation was done twice, and the present data represent the mean of three individual experiments, taken as ± SEM, where **or##*p* < 0.01 and ***or###*p* < 0.001 vs. control.

We tried to find out the effect of SDS-203 on the mobilization and consequent activation of mTORC1 on the lysosomal membrane. For that cells were treated with SDS-203 then processed for colocalization (yellow) evaluation of LAMP1 (green) and mTORC1 (red) by using confocal microscopy. Our results revealed that in control or well-fed MIA PaCa-2 cells LAMP1 and mTORC1 shows colocalization (yellow), while SDS-203 or rapamycin-treated samples showed dispersed fluorescent signals of the respective proteins (Fig. 6A, B). Similar results were confirmed by Co-immunoprecipitation assay (CO-IP) of the SDS-203 treated MIA PaCa-2 cells (Fig. 6C). Further, our results illustrated that SDS-203 treatment failed to overexpress LC3-II and LAMP-1 expression in transiently mTORC1 upregulated pancreatic cancer cells compared to control (Fig. 6D–G). Therefore, above data demonstrates that SDS-203 induces lysosomal biogenesis and its accumulation via mTORC1 inhibition.

### SDS-203 induces lysosomal biogenesis and its enzyme activity through the mTORC1-TFEB pathway

**Figure 7:**
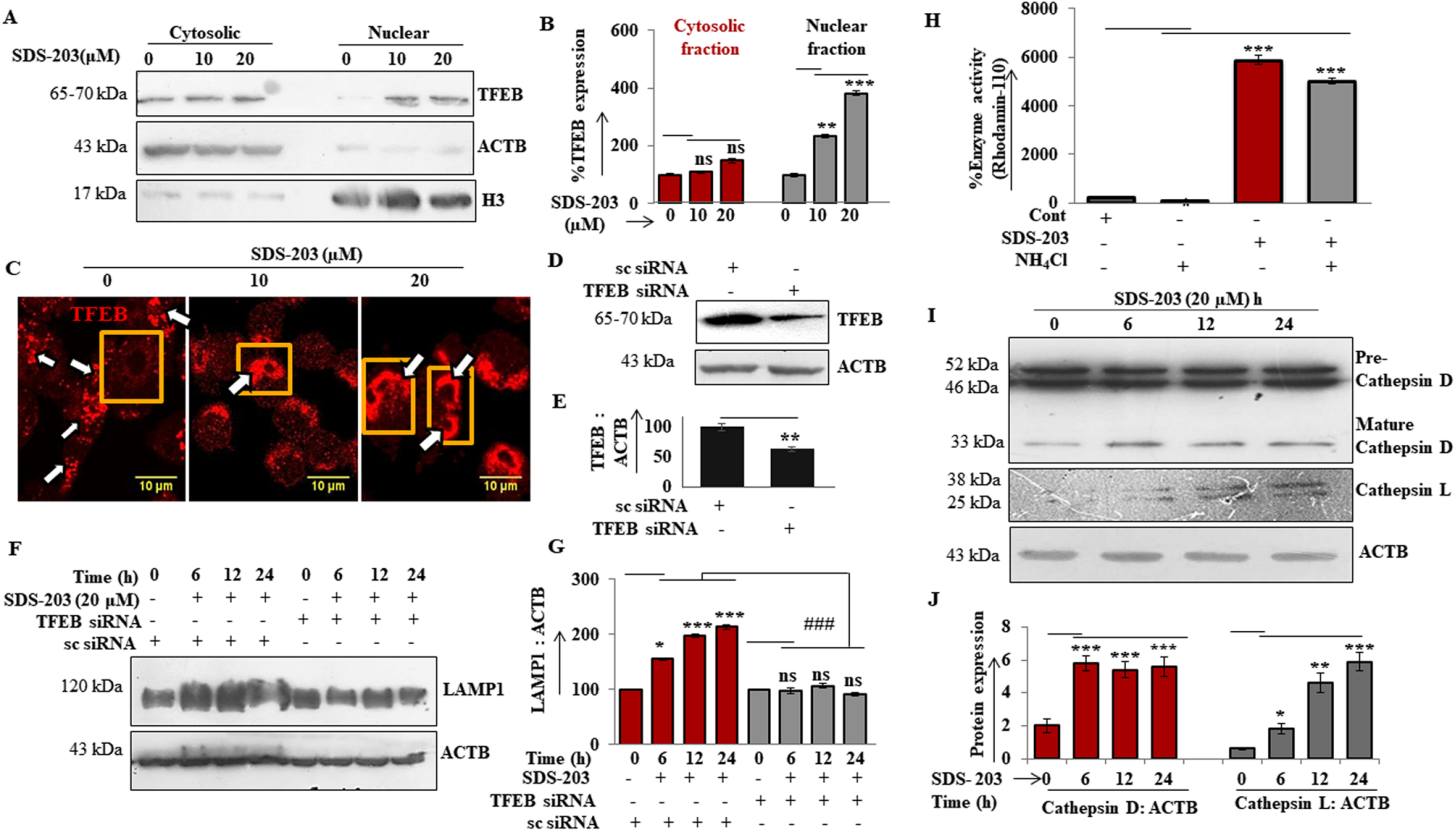
TFEB translocation and lysosomal enzyme activation upon suppression of mTORC1 by SDS-203 treatment. (**A**, **B**) Western blot representation and band quantification of TFEB protein expression in cytosolic and nuclear fraction of MIA PaCA-2 samples treated with SDS-203 (0, 10, 20 µM) for 24 h. (**C**) confocal image representation showing TFEB nuclear translocation upon SDS-203 treatment (0, 10, 20 µM) for 24 h in MIA PaCa-2 cells(**D, E**) Western blot illustration of TFEB knockdown i n MIA PaCa-2 cells and its densitometric quantification. (**F, G**) shows western blot for LAMP1 expression upon SDS-203 treatment in TFEB Knockdown (siRNA) and TFEB scramble g MIA PaCa-2 cells and its densitometric quantification (**H**) Effect of SDS-203 on lysosomal activity: MIA PaCa-2 cells were treated with SDS-203 (20 µM) with or without NH_4_Cl, cell lysates were then subjected to measure lysosomal enzyme activity by analyzing rhodamin110 fluorescence. (**I**) Shows time course western blotting analysis and (**J**) densitometric quantification of lysosomal enzymes- cathepsin D/L in MIA PaCa-2 cells upon SDS-203 treatments. Protein fold change was quantified by using ImageJ software. Error bar indicates ± SEM. *p < 0.05, **p < 0.01, ***or###*p* < 0.001 vs. control.

After confirming that SDS-203 inhibits the localization of mTORC1 on the lysosomal membrane, we further checked its consequence on downstream proteins transcription factor EB (TFEB) related to lysosomal biogenesis, whose subcellular location is controlled by mTORC1 activity^30^. Our immunoblot and immunofluorescence results confirmed that SDS-203 treatment translocates the TFEB into the nucleus of pancreatic cancer cells (Fig. 7A–C).

Moreover, SDS-203 failed to enhance the expression of LAMP1 in TFEB knocked down MIA PaCa-2 cells (Fig. 7D–G). MTT assay revealed that SDS-203 treated TFEB knockdown MIA PaCa-2 cells showed increased survival potential compared to scrambled siRNA treated cells (Supplementary Fig. 4a). To further understand whether SDS-203 induced lysosomal biogenesis was independent of autophagy, western analysis of LAMP1 expression in Beclin1, ATG5, and ATG7 transiently knock out MIA PaCa-2 cells treated with SDS-203 was analyzed. (Supplementary Fig. 4b,c) showed that SDS-203 still maintained the lysosomal biogenesis to several folds in genetically manipulated pancreatic cancer cells compared to sc siRNA-treated cells. Together these results illustrate that SDS-203 translocated TFEB from cytosol to nucleus by inhibiting the activity of mTORC1 which in turn upregulates lysosomal biogenesis independent of autophagy.

Furthermore, we examined the effect of SDS-203 on lysosomal function by checking its effect on cathepsins, important proteases in the lysosome. Fluorescence measurement of Rhodamine 110 (lysosomal enzyme–substrate) analysis demonstrates that SDS-203 upregulated the Rhodamine 110 signal (the activity of lysosomes) to several folds vs. control (Fig. 7H). Western blotting on other hand revealed that expression of enzymes, particularly cathepsin D and cathepsin L was increased in MIA PaCa-2 cells upon SDS-203 treatment (Fig. 7I, J). Using Rhodamine 110, we investigated whether the upregulation of lysosomal enzyme activity by SDS-203 was also mediated by mTORC1 regulation in MIA PaCa-2 cells. Comparative study (Supplementary Fig. 4d) demonstrated that SDS-203 was unable to upregulate lysosomal enzyme activity in mTORC1 upregulated MIA PaCa-2 cells. Enzyme activation potency was further downregulated by using mTORC1 activators like l-leucine or IGF-1 in presence of SDS-203. Above results demonstrate that SDS-203 induces lysosomal biogenesis by increasing the lysosomal enzyme activity resulting in cancer cell death independent of autophagy.

### Addition of late autophagy inhibitors rescued SDS-203 mediated cell death

In order to establish the fact that SDS-203 induced pancreatic cancer cell death by upregulation of lysosomal biogenesis and increased its enzyme activity. We treated MIA PaCa-2 cells with late (bafilomycin A1 or NH_4_Cl) and early (wortmannin or 3-methyladenine: 3-MA) autophagy inhibitors in the presence or absence of SDS-203 and then the viability of these cells were checked by MTT assay (Supplementary Fig. 5a, c; Supplementary Fig. 5b, d). The results clearly illustrate that SDS-203 failed to induce death in MIA PaCa-2 in presence of late autophagy inhibitor, while in the presence of early autophagy inhibitor SDS-203 retained its effect in in ducing cell death. The above data demonstrates that SDS-203 causes lysosomal mediated cell death without any prominat role of autophagy.

### In vivo anticancer activity of SDS -20 3 against Ehrlich ascites car cinoma

**Figure 8:**
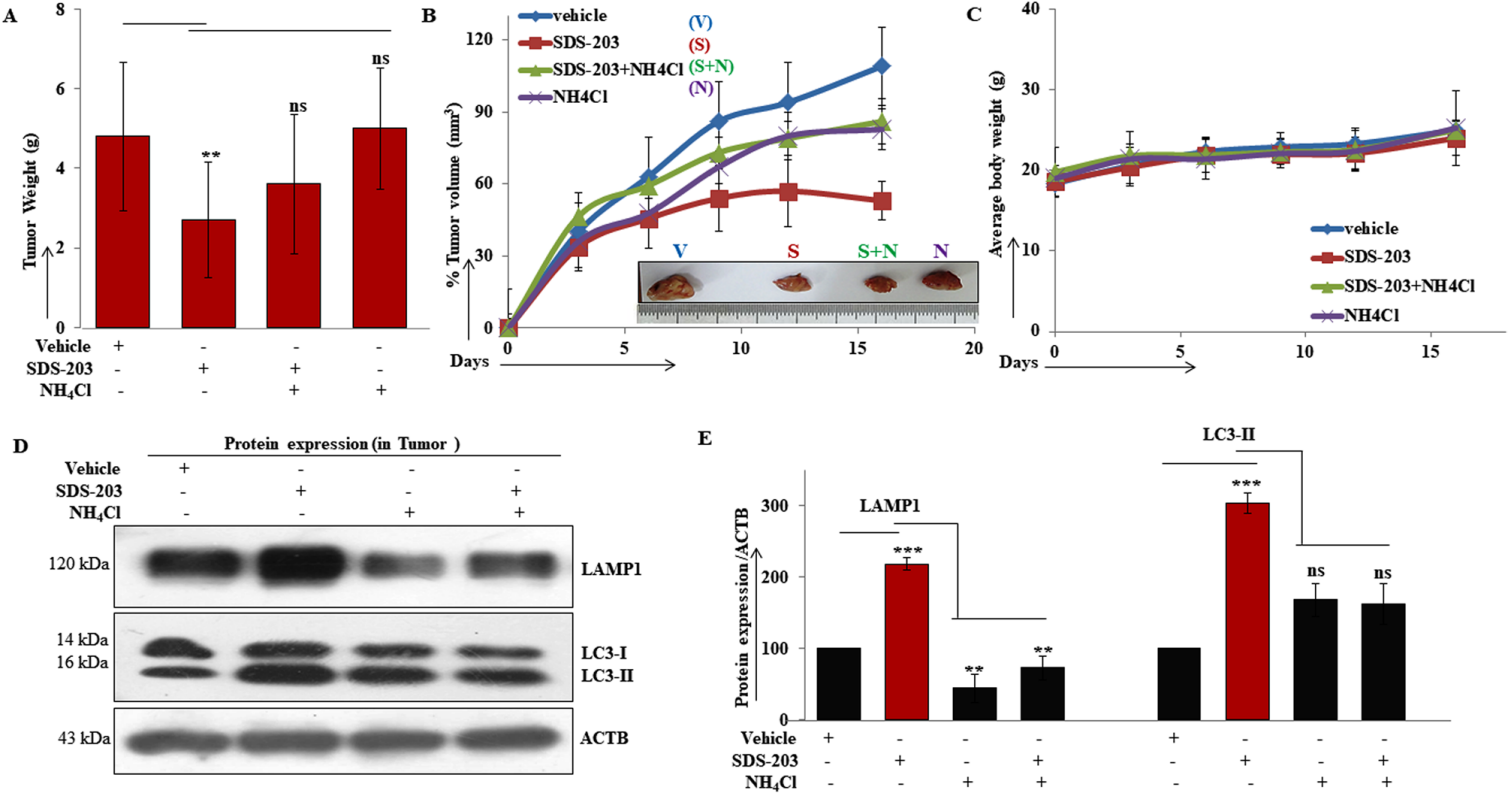
Tumor formation was inhibited upon SDS-203 treatment. Effect of SDS-203 on tumor formation in the four groups of randomized EAC mice model—(vehicle, SDS-203, NH_4_Cl and SDS-203 + NH_4_Cl). (**A**) Represents, tumor weight, (**B**) volume, (**C**) average body weight of each group at various time points. (**D**, **E**) Tumor samples of different groups were lysed for protein extraction which were later resolved and quantified for LAMP1 and LC3-II expression. Data represent the mean ± SD, **p < 0.01, ***p<0.001 vs. vehicle.

The above in vitro lea d was taken for in vivo validation in tumor bearing mice by observing, tumor size (Fig. 8A) and volume (Fig. 8B) that were drastically reduced upon SDS-203 treatment compared to un-treated or NH_4_Cl only treated groups, without affecting the average weight of mice (Fig. 8C). Later equal quantity of proteins extracted from the tumor samples were resolved through western blotting for LAMP1 and LC3-II expression (Fig. 8D, E). Our results confirmed that LAMP1 and LC3-II expressions were overexpressed in SDS-203 treated tumor samples compared to vehicle control or NH_4_Cl only treated tumor samples. Collectively, our in vivo data demonstrated that SDS-203 was equally effective in the upregulation of lysosomes in tumor cells that resulted in the reduction of tumor burden in mice.

### SDS-203 affects multiple targets in pancreatic cancer cells

**Figure 9:**
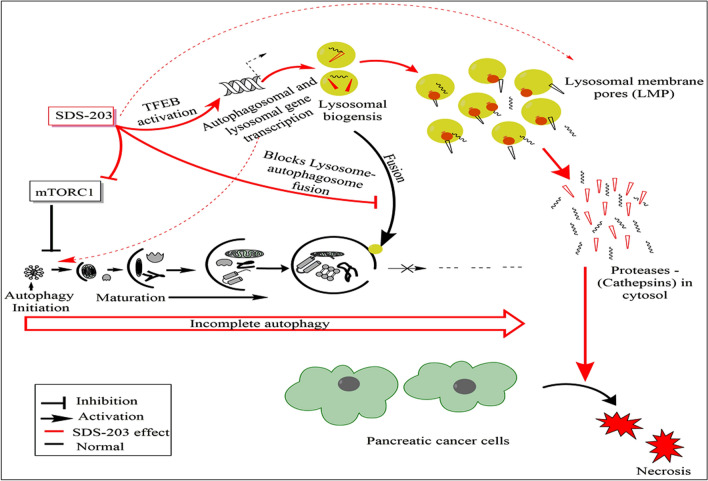
Image representing the effect of SDS-203 on various pathways in pancreatic adenocarcinoma. A schematic diagram represents the potential role of SDS-203 in inhibiting the mTORC1 activity and autophagosome–lysosomal fusion. mTORC1 inhibition translocates the cytosolic TFEB into the nucleus which in turn activates the transcription of lysosomal-related genes resulting in the over synthesis and activation of lysosomal enzymes thereby commencing necrotic death in the aggressive pancreatic cancer cell MIA PaCa-2. This representation has been made with the help of the software ChemBioDraw Ultra 14.

The pictorial representation (Fig. 9) shows the mechanism of pancreatic cancer cell death induced by SDS-203. Inhibitory effect of SDS-203 on mTORC1 led to the activation of incomplete autophagy and TFEB nuclear translocation, triggering strong lysosomal biogenesis. Accumulation of lysosomes ultimately leads to the culmination of pancreatic cancer cells. The graphical image was drawn by using the software ChemBioDraw Ultra 14.

## Discussion

The majority of the current anticancer therapies are positioned to target apoptotic machinery to induce non-pathological cell death in cancer cells^31^. However, the development of resistance during the course of treatment remains a monumental challenge for successful and effective treatment^32^. During treatment, defects in apoptotic cell death occur due to dysregulation at various programmed steps, which may lead to acquired treatment resistance^33^. Hence the identification of small-molecule agents targeting cancer cells through non-apoptotic machinery is desirable to achieve an effective and lasting therapeutic response^34,35^. In this study, we reported that small-molecule SDS-203 induced cell death in most aggressive pancreatic cancer cells through the non-apoptotic route by up-regulating lysosomal biogenesis and its proteasomal enzyme activity. The functional upregulation of lysosome machinery in MIA PaCa-2 cells upon SDS-203 treatment is mediated via inhibition of mTORC1, as reported earlier by our group^26^. mTORC1 negatively regulates lysosomal function by directly phosphorylating TFEB on the lysosomal membrane, thus allowing interaction between TFEB and 14-3-3 keeping TFEB sequestered in the cytoplasm^36,37^. SDS-203 mediated inhibition of mTORC1 led to the disengagement of TFEB from 14-3-3 allowing TFEB to translocate into the nucleus where it causes activation of various genes associated with autophagy and lysosomal biogenesis. We demonstrated that SDS-203 mediated inhibition of mTORC1 also triggered an early autophagy response in cells as observed by the expression of LC3-II and ATG5. Surprisingly our data revealed an increase in the levels of an adapter protein p62 during the course of treatment, thus indicating an incomplete autophagic response caused by SDS-203.

To better understand the role of SDS-203 triggered autophagy in cell death, we attempted to use both early and late autophagy inhibitors during the course of SDS-203 treatment. The use of early autophagy inhibitors 3-MA and wortmannin were unable to rescue the SDS-203 mediated cell death in MIA PaCa-2 cells. However, the addition of late autophagy inhibitors bafilomycin A1 and NH_4_Cl showed considerable protection against the effect of SDS-203. It was interesting to find that under similar conditions addition of late autophagy inhibitors did not alter the expression of LC3-II and p62 when compared to SDS-203 treatment alone, showing defective autophagy flux or incomplete autophagy. On the other hand, treating starving cells with SDS-203 or co-treatment with rapamycin showed enhanced expression of LC3-II and p62, indicating SDS-203 also inhibited autophagy flux.

To further apprehend the mechanism of SDS-203 mediated cell death, we tried to understand the role of lysosomal machinery that gets activated during the course of treatment. Microscopic examination and immunoblotting experiments for lysosomal marker protein LAMP1 indicated its enhanced expression in cells treated with SDS-203. Correspondingly, flowcytometric analysis using lysotracker red further supported enhanced lysosomal biogenesis in SDS-203 treated cells. Additionally, hydrolytic lysosomal enzyme activity in SDS-203 treated cells was measured by using nonradioactive fluorescent probe Rhodamine 110 clearly showed that enzyme activity was several folds higher as compared to control or NH_4_Cl treated cells, thereby supporting the role of lysosomes in treating cancer cells. Similarly, the expression of lysosomal proteases Cathepsin D and Cathepsin L were upregulated in SDS-203 treated cells that play a vital role in protein degradation and cell death.

TFEB is the major transcriptional regulator of autophagosomal/lysosomal proteins and its translocation into the nucleus is controlled by its phosphorylated state regulated by mTORC1^38^. Our study found that SDS-203 treatment facilitated the nuclear translocation of TFEB via early inhibition of mTORC1. To further understand the role of TFEB mediated lysosomal cell death under SDS-203 treatment, we performed gene knockdown of TFEB by using siRNA, and observed that the expression of LAMP1 was considerably downregulated. We also observed that TFEB knockdown significantly rescued cell death induced by SDS-203 treatment, thereby showing that mTOR/TFEB/LAMP1 cascade plays a dominant role in lysosome mediated non-apoptotic death in MIA PaCa-2 cells. In vitro observations of SDS-203 mediated lysosomal cell death were further augmented by the outcome of results observed in mice tumor models, where lysosomal quencher NH_4_Cl treatment subdued the SDS-203 induced tumor growth. Protein analysis of tumor samples excised from mice has further demonstrated that SDS-203 significantly enhanced the lysosomal marker protein LAMP1, indicating the role of SDS-203 mediated lysosomal upregulation in tumor regression without affecting the overall health of the mice. In summary, we found that SDS-203 treatment facilitated the nuclear translocation of TFEB via early inhibition of mTORC1 leading to lysosomal mediated cell death.

## Conclusion

Our studies have demonstrated that small molecule SDS-203 induced non-apoptotic death in highly aggressive pancreatic cancer cells. Further, mechanistic understanding revealed that SDS-203 triggered initial autophagic response, resulting in excessive lysosomal accumulation with enhanced proteolytic enzymatic activities causing cell death. These studies remarked the prospective use of SDS-203 against cancers with dysfunctional apoptotic machinery and warrants further pre-clinical studies.

## Materials and methods

### Chemicals, reagents and antibodies

The chemicals and reagents used in our experiments were: bafilomycin A1(Baf, Sigma, #19-148) chloroquine (CQ, Sigma, C6628), rapamycin (Sigma, #R8781) cathepsin B substrate, GFP-LC3 (plasmid, CST), and mTORC1 (WT plasmid, addgene #11546), DMSO (#D2650), 2′,7′-dichlorofluorescin diacetate (H_2_DCFDA) (Sigma, #35845), EDTA (Sigma, #E9884), Propidium iodide (Sigma, #P4170), Insulin-like growth factor (IGF-1, Sigma, #I3769), L-Leucine (Sigma, #L8000), acridine orange dye (Sigma, #235474), LysoTracker™ Red DND-99 (ThermoFisher, #L7528), Phosphate buffer saline (Sigma, PBS, #P5493), 3-(4,5-dimethylthiazol-2-yl)-2,5-diphenyltetrazolium bromide (MTT, Sigma, #M2128), phenylmethylsulfonyl fluoride (PMSF, Sigma, #P7626), Sodium orthovanadate (Sigma, #S6508), annexin V and propidium iodide (PI) (Santa Cruz Biotechnology, #sc-4252), fixer (Sigma, #P7167), developer (Kodak, Sigma, #DA163, #P6557), protease inhibitors cocktail (Sigma, #P8340), PVDF membrane (0.2 µm, BIO-RAD#162-0177), protein assay kit (BIO-RAD, #500-0002), chemiluminescences film (Bioscience, #28906035), protein ladder (BIO-RAD, #161-0374), Amersham™ Hypercassette™ (#RPN11644).

The antibodies used in our experiments were: **Cell Signaling Techonology** (CST): ATG7 (#8558P), p-mTOR (#2971P, #2974S), m-TOR(#2983S) PARP ( #9542SS), ATG5 (#8540S), p70S6K (#9205, #9234P), p4EBP (#9455P, #2855P), anti-p62 antibody (#5114, #8025S), ATG7 siRNA (#6604), ATG5 siRNA (#6345), Beclin-1 siRNA (#6222), beclin-1antibody (#3495S), Caspase-3 (#9662S) **Sigma-Aldrich**: Anti-LC3B antibody (#L7543), p62 (#P0067), Monoclonal anti-β-Actin antibody (#A3854), anti Rabbit IgG-peroxidase antibody (A9169) **Santa Cruz** **Biotechnology**: cathepsin D antibody (Santa Cruz, SC-6486), cathepsin L antibody (Santa Cruz, SC-6498), TFEB (H-125) SC-48784, LAMP1 (H4A3) SC-2001 Mouse monoclonal.

### Cell culture

MIA PaCa-2 cells were obtained from the American Type Culture Collection (ATCC). Cell lines were maintained in DMEM (Sigma, D1152) containing 10% fetal bovine serum (FBS) in a standard cell incubation conditions—humidity around 95%, 5% CO_2_ at 37 °C.

### MTT assay

MTT (3-(4, 5-Dimethylthiazol-2-yl)-2, 5-diphenyltetrazolium bromide) assay was used to check cell viability. MTT solution prepared in phosphate buffer saline (PBS) with the concentration (5 mg/mL) was incubated with pancreatic cancer cells treated with SDS-203 for 2 h before termination of the experiment._._ Live cells change MTT to formazan crystals which were dissolved in dimethyl sulfoxide (DMSO) giving a purple color solution. Intensity of the color was measured by using a microplate reader (TECAN) with absorbance reading at 570 nm.

### Flowcytometry

SDS-203 treated pancreatic cancer cells were incubated with various dyes and the cell population was analyzed using flowcytometry. Briefly, 0.2 × 10^4^ MIA PaCa-2 cells were seeded in 6-well plates and later treated with SDS-203- in time (0, 6, 12, 24 h) or concentration-dependent manner (0, 5, 10, 20, 30 µM). Before termination of the experiment cells were stained with various dyes—Annexin V FITC/PI or DCFDA or Lysotracker red DND in incomplete media for 20 min each. Samples were later washed twice with PBS then trypsinized and resuspended for analysis through flowcytometry (BD FACS Calibur). 10,000 live events /sec were taken for data collection.

### Protein isolation, quantification, western blotting and immunoprecipitation

At the end of the designated treatments, cells were lysed by RIPA or M2 lysis buffer and collected for protein estimation, each steps were done at 4 °C. Following that equal amount of protein from each samples were resolved by SDS-PAGE and transferred onto polyvinylidene difluoride (PVDF) membrane (Bio-Rad, 162-0177). Later PVDF membrane was blocked with 5% non-fat milk and cut into strips (based on molecular weight; using Bio-Rad protein standard 10–250 kDa) prior to hybridization with target primary antibodies and HRP^+^ tagged secondary antibodies. Western signaling was detected by using chemiluminescence Horseradish Peroxidase (HRP) substrates and the signals were captured on X-ray film. The density of the various bands in the western blot was quantified using ImageJ software.

### Acridine orange staining

Acid vesicular organelles (AVO) formation is a characteristic feature of autophagy induction. To detect the formation of AVO, acridine orange was used. Accumulated AO in acid compartments gives bright red fluorescence (exc = 488 nm laser). 2 × 10^4^ cells were seeded in 6 well plates and then treated with SDS-203 in a time-dependent manner (0, 6, 12, 24 h). 15 min before termination AO (1 µg/mL) was added, post staining cells were washed with PBS and taken for microscopy. Data were analyzed by using ImageJ software.

### Transfection

GFP-LC3 (plasmid), TFEB siRNA and mTORC1 (WT plasmid) were used to transiently transfect pancreatic cancer cells, later SDS-203 was treated to these transfected cells. MIA PaCa-2 cells were seeded in 6 well plates and allowed to grow up to 70% confluency and then incubated with the plasmid along with transfection reagent, (Fugene kit based transfection reagent; Promega FuGENE HD Transfection Reagent #E2311) in an incomplete media for 14 h. Desired concentration of SDS-203 was later treated to the cells and its effect on the expression of the above-mentioned targets was calculated.

### Immunofluorescence and confocal microscopy

Expression of various proteins like LC3-II, mTORC1, LAMP1, GFP-LC3 and intensity of lysotracker DND-red or DCFDA dye in pancreatic cancer cells upon SDS-203 treatment were analyzed by using fluorescence microscopy. 0.2 × 10^4^ MIA PaCa-2 cells were allowed to grow on coverslips in 6-well plates, after selected treatment of SDS-203 for particular time points were either incubated with dyes for 15 min before termination or fixed with 4% paraformaldehyde (PFA) for 15 min then permeabilized with (0.02% of Triton-X-100 for 5 min) and later blocked by using blocking buffer (3%BSA + 0.01% Triton-X-100 in PBS for 1 h). Blocked samples were then incubated with primary antibody (mentioned dilution) overnight at 4 °C. Dye treated or GFP-LC3 transfected samples were washed and mounted on glass slides. While antibody incubated samples were washed and incubated with anti-Alexa Fluor secondary antibody for 2 h at room temperature, meanwhile samples were washed 3 times with PBS and mounted on glass slides. Mounted samples were then visualized under confocal microscopy. Ten different fields of each sample were taken and final results were taken as an average mean of each sample. Data interpretation was done by using ImageJ software

### Electron microscopy

MIA PaCa-2 cells were seeded in complete DMEM high glucose and treated with 20 μM SDS-203 and rapamycin 200 nM for 24 h, and pelleted down at 1800 rpm for 10 min. Cell pellets were fixed with 2.5% glutaraldehyde at 4 °C for 1 h, then post-fixed with 1% OsO_4_ for 1 h. Samples were later dehydrated from lower to higher graded ethanol solutions and then embedded in Epon 812^39^. Semi-thin sections of samples were taken and stained with 1% toluidine aqueous blue at 40 °C and were observed with a Vanox light microscope (Olympus, Tokyo, Japan). Ultrathin sections of 60–90 nm were taken with an LKB ultramicrotome (LKB, Bromma, Sweden), which were collected on copper grids and stained with uranyl acetate and lead citrate. Stained sections were examined with a JEOL JEM 1400CXII electron microscope at 80 kV. To evaluate ultrastructural alterations, ~ 100 cells per sample were examined from two independent experiments.

### Measurement of lysosomal enzyme (CTSB-CTSL) activity

Enzyme activity of lysosome was done by a nonradioactive method by using rhodamine-110 following protocol^36,37^. Rhodamine 110 (R110) is a sensitive and selective probe for assaying proteases in the cell lysate. This dye contains a fluorogenic substrate called Rhodamine. The substrate can be used to measure lysosomal enzyme activity in cell extracts or purified enzyme preparations using a fluorescence microplate reader. Treated cancer cell samples (SDS-203, NH_4_Cl or SDS-203 + NH_4_CL) were lysed by M2 buffer 20 mM Tris at pH 7, 0.5% NP-40, 250 mM NaCl, 3 mM EDTA, 3 mM EGTA, 2 mM dithiothreitol, 0.5 mM phenylmethylsulfonyl fluoride, 20 mM glycerol phosphate, 1 mM sodium vanadate and protease inhibitor cocktail for protein extraction. Protein estimation was done in order to incubate equal amount of (25 µg) protein from each sample with 50 µM of Rhodamine110, in 100 µL cell-free system buffer (10 mM HEPES–NaOH pH 7.4, 68 mM sucrose 220 mM mannitol, 2 mM NaCl 2.5 mM KH_2_PO_4_, 0.5 mM EGTA, 2 mM MgCl_2_, 5 mM pyruvate, 0.1 mM PMSF and 1 mM dithiothreitol) in 96 well plate for 1 h at 37 °C. Reading was taken at an exc/emi wavelength = 380/460 nm by using fluorometry (Tecan Spectra Fluor Plus). Activity was represented as a percentage of fluorescence intensity compared with the control group.

### In vivo tumor mice model

The animals were housed under standard husbandry conditions: 24 ± 2 °C temperature, 15–20 complete fresh air changes per hour and 50–60% relative humidity as per guide for the care and use of laboratory animals. Animals were fed with a standard pellet diet (M/S Ashirwad Industries, Chandigarh, India) and autoclaved water was given ad libitum. Approval of the Institutional Animal Ethics Committee, CSIR-Indian Institute of Integrative Medicine, Jammu was sought for the study and number of animals used in all the experiments. SDS-203 was taken for in vivo anticancer assessment against murine tumor model and NH_4_Cl was taken as a negative control. Swiss albino mice (18–23 g) under optimum laboratory conditions were injected with Ehrlich Tumor cells (EAC), grafted from 8 to 10 days old ascites tumor-induced Swiss albino mice. On day zero 1 × 10^7^ EAC cells were injected intraperitoneally, later tumor-induced animals were categorized randomly into four test groups, with seven animals per group. The first test group was administrated with normal saline (0.9% i.p.) which act as vehicle control. Another group was treated with SDS-203 (25 mg/kg i.p), and the remaining two groups were treated with or without SDS-203 in presence of NH_4_Cl (20 mg/kg i.p). Treatment was followed up to nine consecutive days; tumor assessment was done on day 12. Tumor measurements included size, weight and volume, and were taken from different groups. Some tumor samples from each group were frozen for protein extraction, which was later done by using tissue lysis buffer and homogenizer. All animal experimental procedures were carried out following the ethical guidelines for the use of animals in experiments and were conducted in compliance with the Committee for the Purpose of Control and Supervision of Experiment on Animals (CPCSEA) and the ARRIVE guidelines. All experiments were approved by the animal house CSIR IIIM. The use of experimental animals in this study was approved by the Ethics and Institutional Animal Care and Use, Committees of Council of Scientific and Industrial Research-Indian Institute of Integrative Medicine (CSIR-IIIM).

### Ethical approval and consent to participate

The use of experimental animals in this study was approved by the Ethics and Institutional Animal Care and Use, Committees of Council of Scientific and Industrial Research-Indian Institute of Integrative Medicine (CSIR-IIIM) following guidelines of the Committee for the Purpose of Control and Supervision of Experiment on Animals (CPCSEA). I confirm that all methods were carried out "in accordance with relevant guidelines and regulations. I confirm that study was carried out in compliance with the ARRIVE guidelines.

### Institutional publication number

CSIR-IIIM/IPR/00218.

## Supplementary Information

Supplementary Figures.

## Data Availability

The datasets used and/or analyzed during the current study are available from the corresponding author on reasonable request.
