# Author Correction: Activation of lysosomal mediated cell death in the course of autophagy by mTORC1 inhibitor

**DOI:** 10.1038/s41598-022-12521-w

**Published:** 2022-05-24

**Authors:** Sameer Ullah Khan, Anup Singh Pathania, Abubakar Wani, Kaneez Fatima, Mubashir Javed Mintoo, Baseerat Hamza, Masroor Ahmad Paddar, Wadhwa Bhumika, Loveleena Kour Anand, Mir Shahid Maqbool, Sameer Ahmad Mir, Jaspreet Kour, Vunnam Venkateswarlu, Dilip Manikrao Mondhe, Sanghapal D. Sawant, Fayaz Malik

**Affiliations:** 1 Pharmacology Division, CSIR-Indian Institute of Integrative Medicine, Sanat Nagar, Srinagar, Jammu and Kashmir 190005 India; 2 Academy of Scientific and Innovative Research (AcSIR), Ghaziabad, Uttar Pradesh 201002 India; 3 Medicinal Chemistry, CSIR-Indian Institute of Integrative Medicine, Jammu, India

Correction to: *Scientific reports* 10.1038/s41598-022-07955-1, published online 23 March 2022

The original version of this Article contained an error in the Figure 1(E) label, where the molecular weight of Caspase-3 (32, 20 kDa) and PARP-1 (116, 89 kDa) were inadvertently switched.

**Figure 1:**
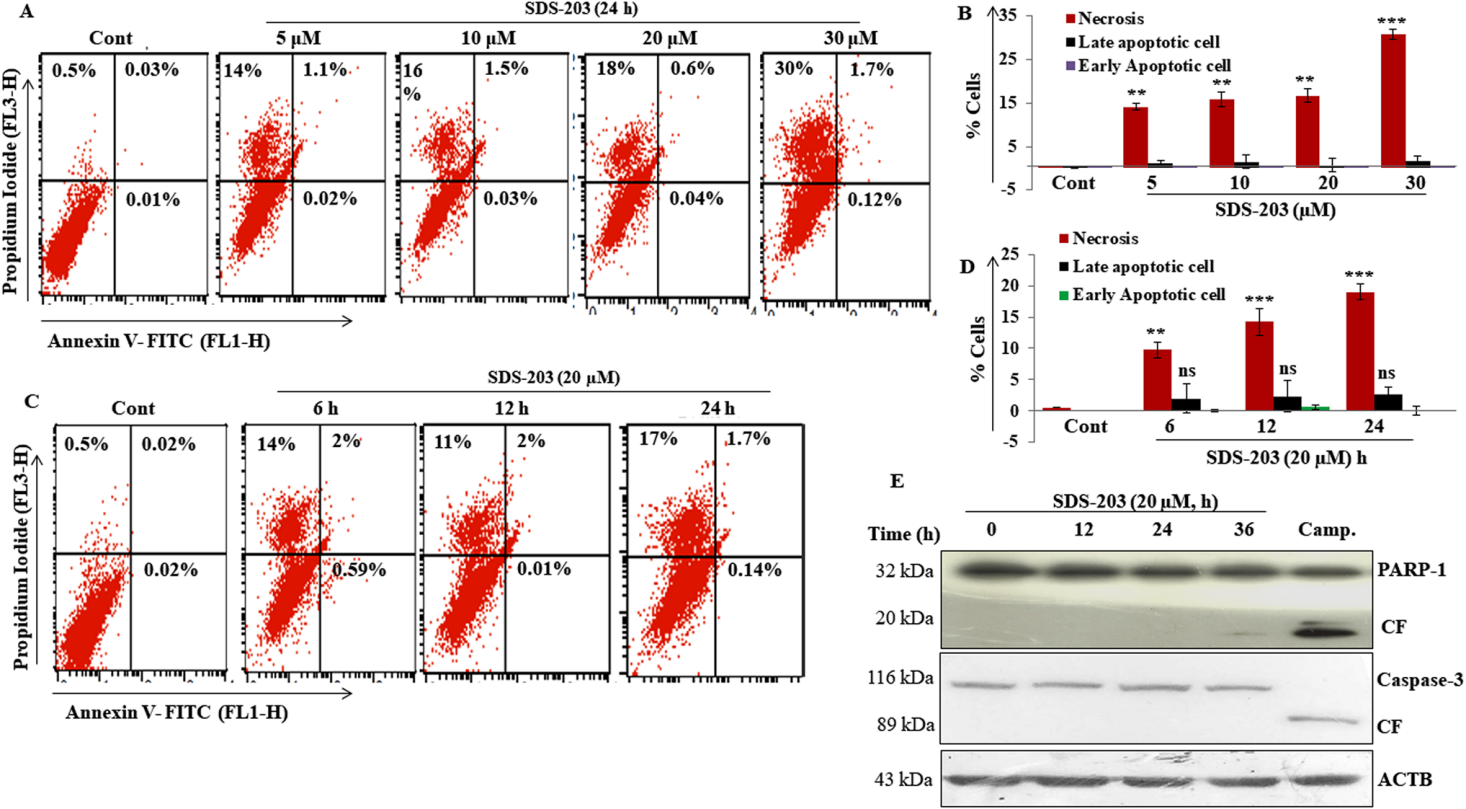
SDS-203 decreases cell viability by the non-apoptotic way in pancreatic cancer cell line MIA PaCa-2. (**A**–**D**) MIA PaCa-2 cells were incubated with different concentrations of SDS-203 (5, 10, 20, 30 μM) for 24 h or single concentration (20 μM) for various time points (0, 6, 12, 24 h) then cells were stained with Annexine-VFITC and PI (1 μg/mL) followed by quantification of apoptotic cells by flowcytometry (**E**) MIA PaCa-2 cells were treated with SDS-203 (20 μM) in a time-dependent manner (0, 12, 24, 36 h) and protein expression of cleaved PARP1 and cleaved Caspase3 were determined by western blotting, camptothecin (1 μM) was taken as a positive control. Each experiment was repeated three times. *p*-value represents: **p < 0.01; ***p < 0.001vs. control.

The original Figure 1 and accompanying legend appear below.

The original Article has been corrected.

