# Supplementary material for: Activation of lysosomal mediated cell death in the course of autophagy by mTORC1 inhibitor
Source: Sci Rep. 2022 Mar 23;12:5052. doi: 10.1038/s41598-022-07955-1 (PMC8943151; doi:10.1038/s41598-022-07955-1)

Supplementary information

Title: Activation of lysosomal mediated cell death in the course of autophagy by mTORC1 inhibitor.

Running title: Pancreatic cancer death by Lysosomes

Author list: *Sameer Ullah Khan*[,1,2,@], *Anup Singh Pathania*[1,2,@], *Abubakar Wani*[1,2,$], *Kaneez Fatima*[1,2,$], *Mubashir Javed Mintoo*[1,2], *Baseerat Hamza*[1], *Masroor Ahmad Paddar*[1,2], *Wadhwa Bhumika*[1,2], *Loveleena Kour Anand*[1,2], *Mir Shahid Maqbool*[1,2], *Sameer Ahmad Mir*[1,2], *Jaspreet Kour*[3], *Vunnam Venkateswarlu*[3], *Dilip Manikrao Mondhe*[1], *Sanghapal D Sawant*[3], *Fayaz Malik*[1,]*.

Supplementary Fig. 1

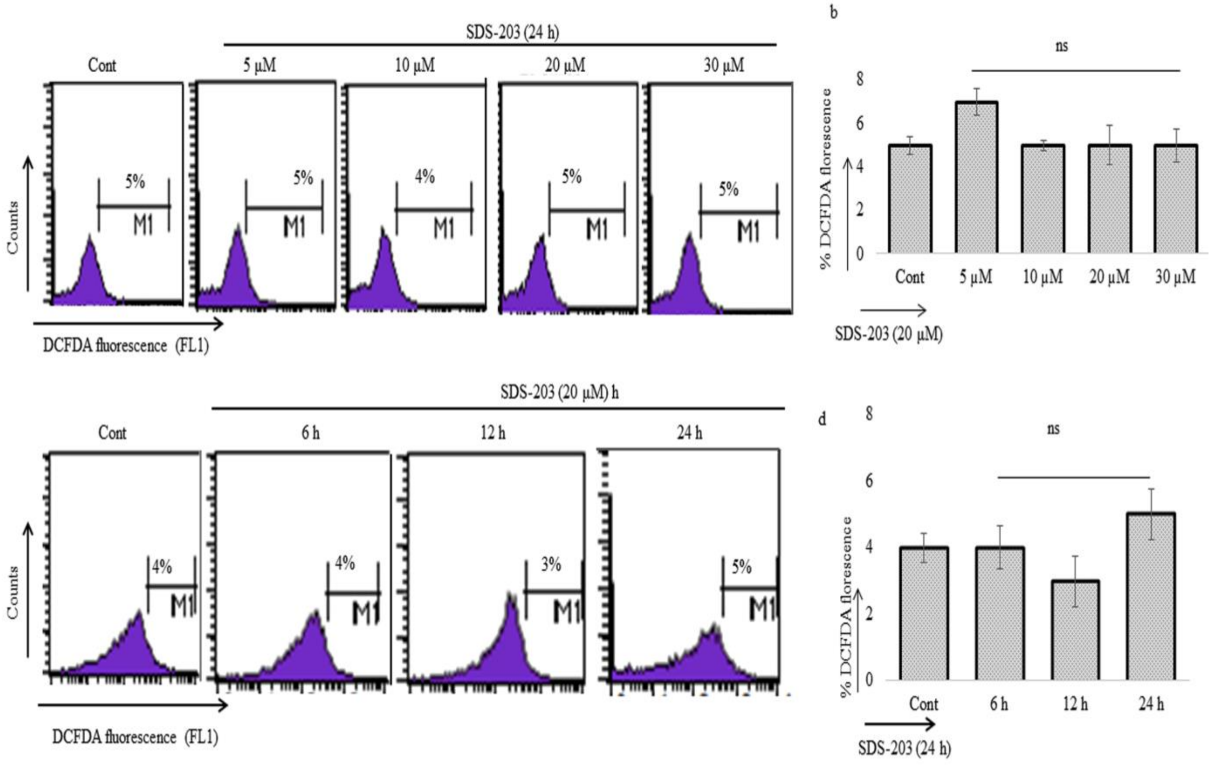

Supplementary Figures 1. **SDS-203 failed to upregulate reactive oxygen species in pancreatic cancer cells.** (a, b) SDS-203 treatment in concentration and time-dependent manner was harvested for ROS level determination by using ROS detection dye (DCFDA) and flowcytometry.

Supplementary Fig. 2

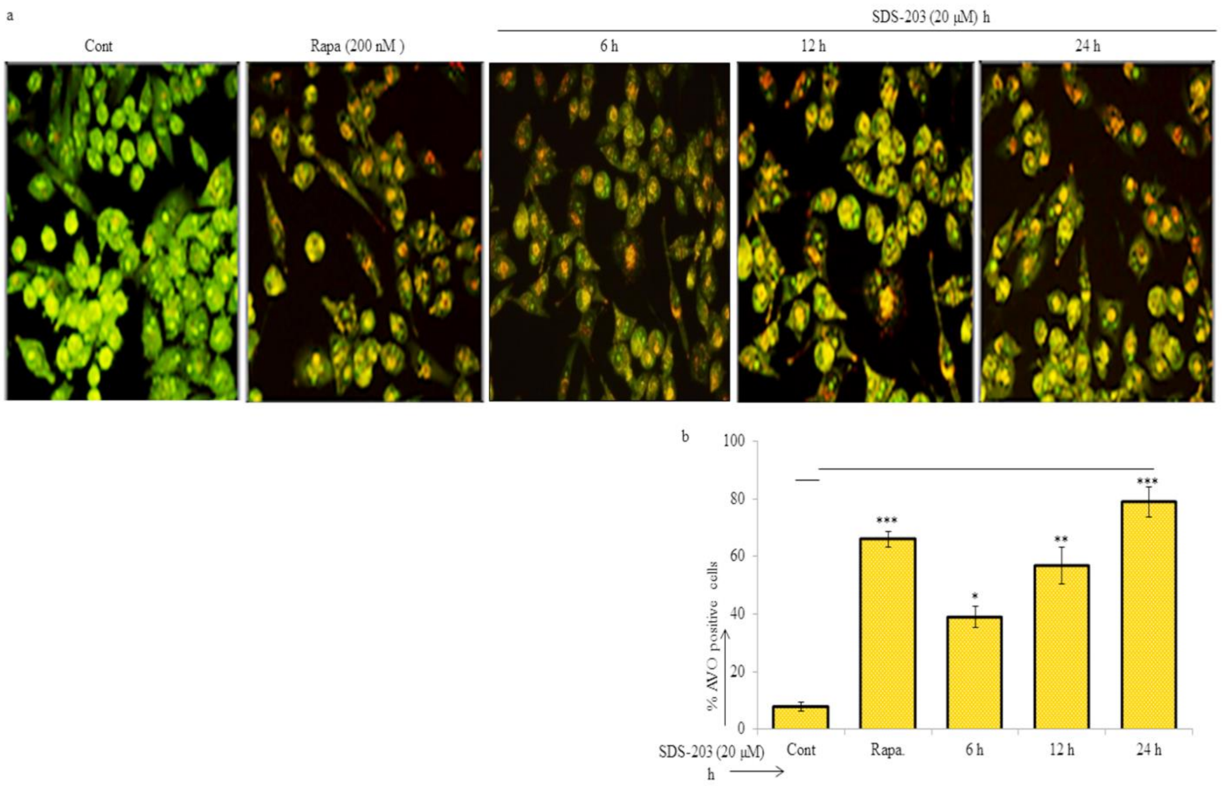

Supplementary Figures 2. **SDS-203 upregulates AO signal in MIA PaCa-2 cells.** Cells incubated with SDS-203 (20 µM) for 6, 12, 24 h or rapamycin (200 nM) later stained with AO (1 µg/mL) samples taken for microscopic imaging (a), represent AO fluorescence images (b). quantification graph represents data of three individual experiments. Data represent the mean±SD **p*<0.05, ***p<0.01*, ****p<0.001*

Supplementary Fig. 3

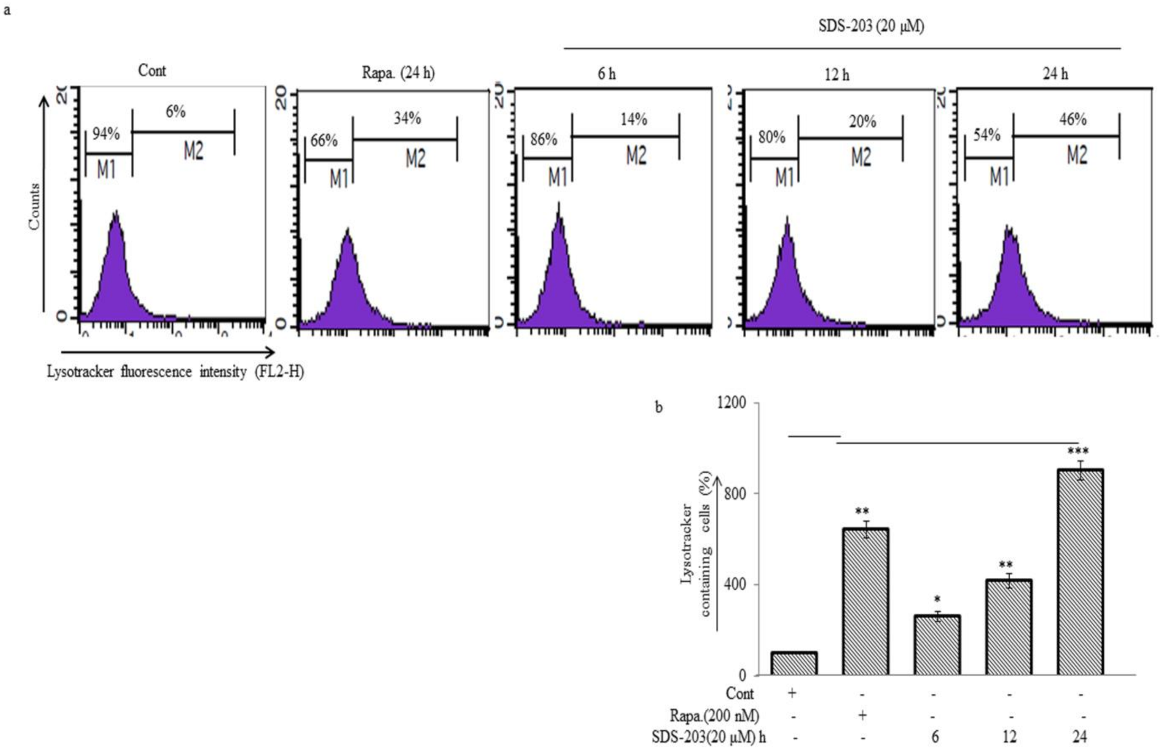

Supplementary Figures 3. **SDS-203 increases lysosomal number in human pancreatic cancer cells**. (a) MIA PaCa-2 cells were exposed to SDS-203 (20 μM) for 0, 6, 12 and 24 h or with rapamycin then stained with 50 nM LysoTracker Red, later samples were analyzed by flowcytometer (b) quantitative analysis of LysoTracker-positive cells. Results shown are representative of at least three independent experiments, the mean±SD *p<0.5, **$p<0.1$, ***$p<0.001$

Supplementary Fig. 4

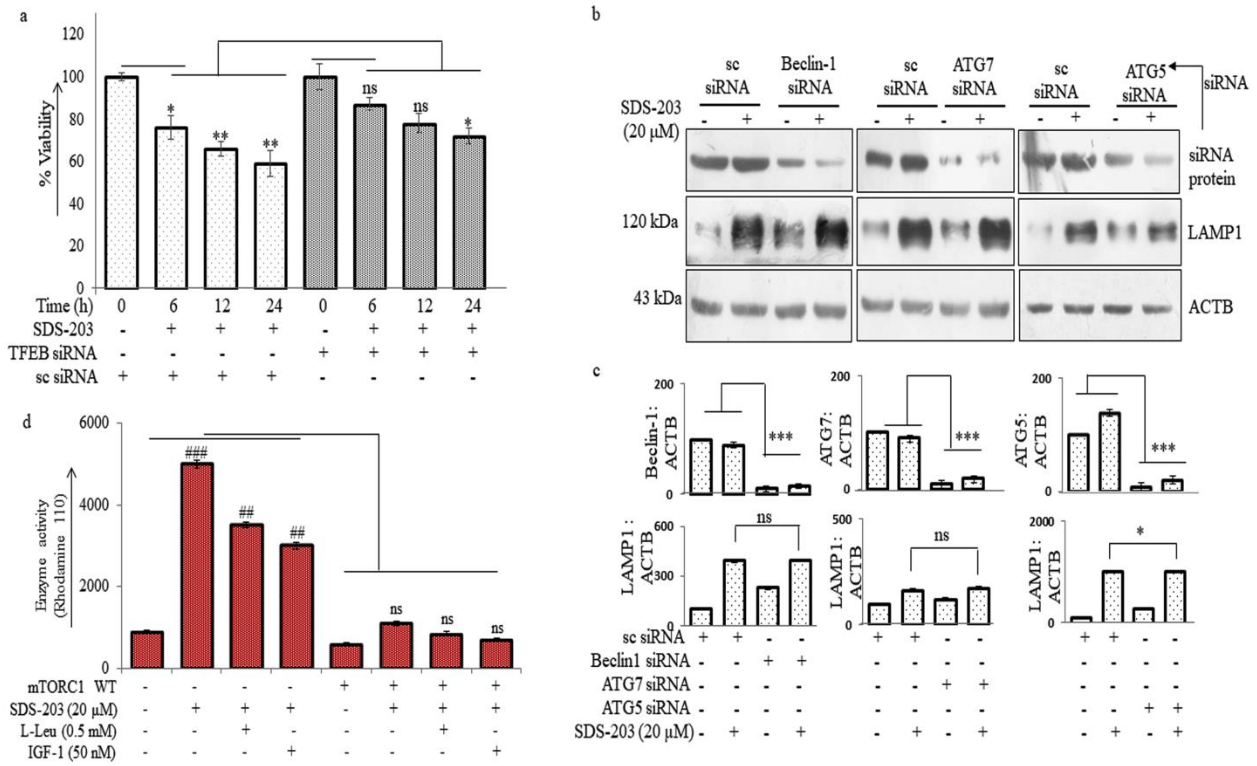

Supplementary Figures 4. **Survival capacity of TFEB knockdown MIA PaCa-2 cells increased in presence of SDS-203 and was independent of autophagy** (a) cell viability of transiently transfected TFEB siRNA or sc siRNA MIA PaCa-2 cells upon SDS-203 (20 µM) for 0, 12, 24, 36 h. Survival percentage was verified by MTT assay. (b) immunoblot representation of important autophagic gene (ATG5 or ATG7 or Beclin-1) in MIA PaCa-2 cells which were transiently transfected with respective siRNAs or sc siRNA. Later effect of SDS-203 (20 µM) on the expression of LAMP1 was validated (c) comparative quantification analysis of protein expression was done by using Image J software. (d) enzyme activity in vehicle-treated or mTORC1 upregulated MIA PaCa-2 cells upon SDS-203 (20 µM) treatment in presence or absence of IGF1 (50 nM) or L-leucine (0.5 mM) for 24 h Results illustrated here are representative of at least three independent experiments, the mean±SD *p<0.05, ***p<0.01*, ****p<0.001*

Supplementary Fig. 5

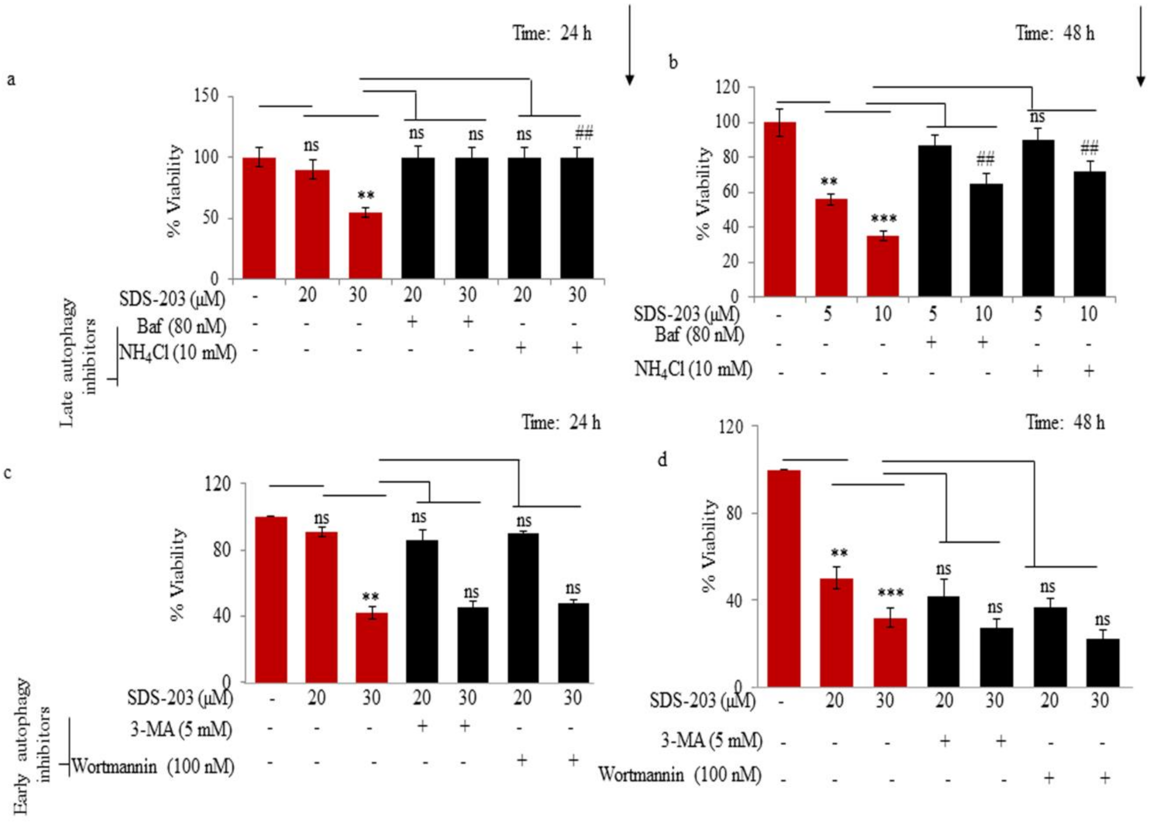

Supplementary Figure 5. **SDS-203 induced death was rescued by late autophagy inhibitors.** (a, b) viability of pancreatic cancer cells treated with SDS-203 (20 and 30 µM) in presence or absence of late or early autophagy inhibitor bafilomycin A1 (80 nM), NH_4Cl (10 mM) or 3MA (5 mM), Wortaminin (100 nM) for 24 and 48 hours respectively. Results shown here are the mean average of three experiments. ±SEM *p<0.05, **<0.01

Raw Data

Main Figure

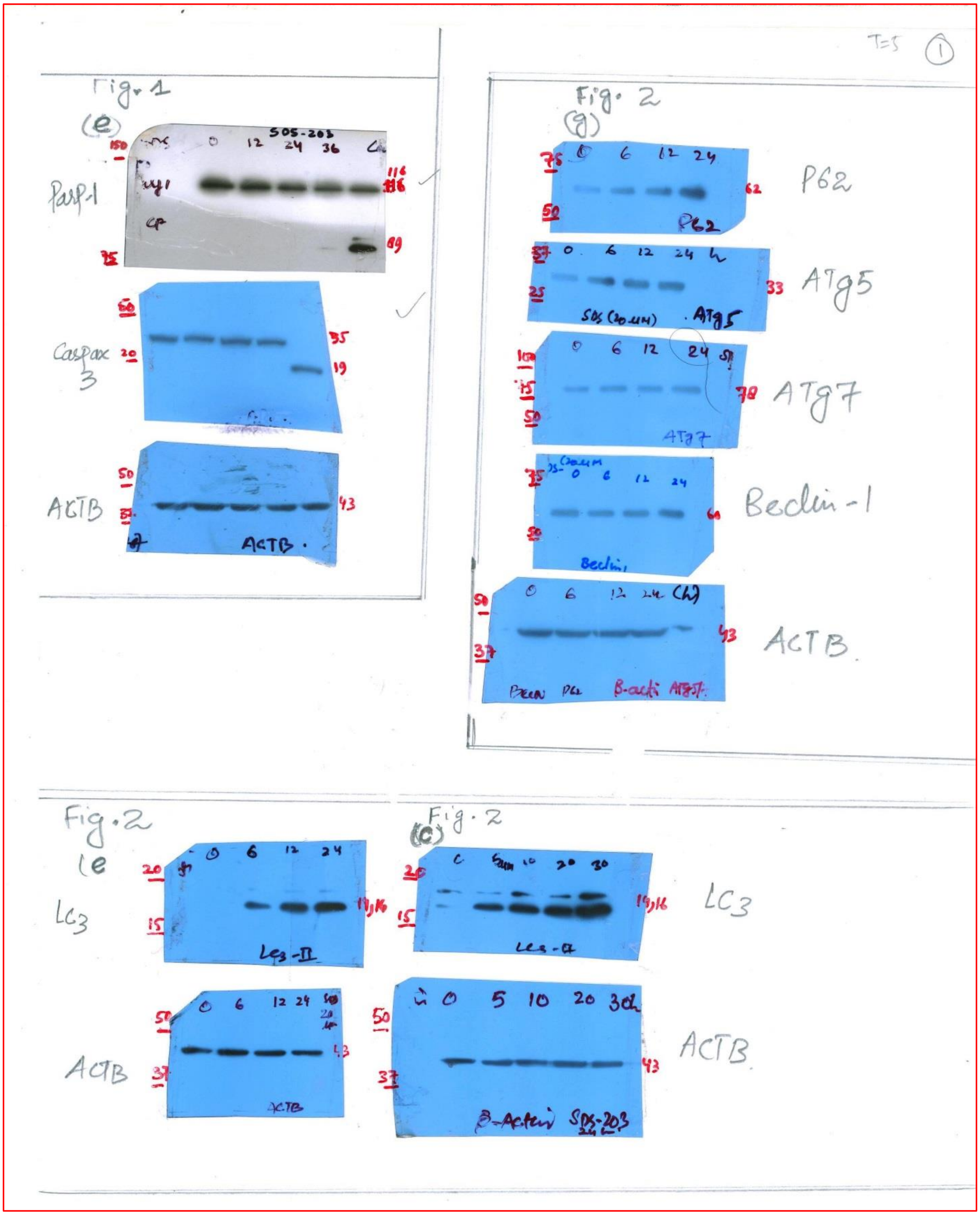
Fig. 1
(e)
Parp-1
Caspase 3
ACTB
Fig. 2
(g)
P62
ATg5
ATg7
Beclin-1
ACTB
Fig. 2
Fig. 2
(c)
LC3
ACTB

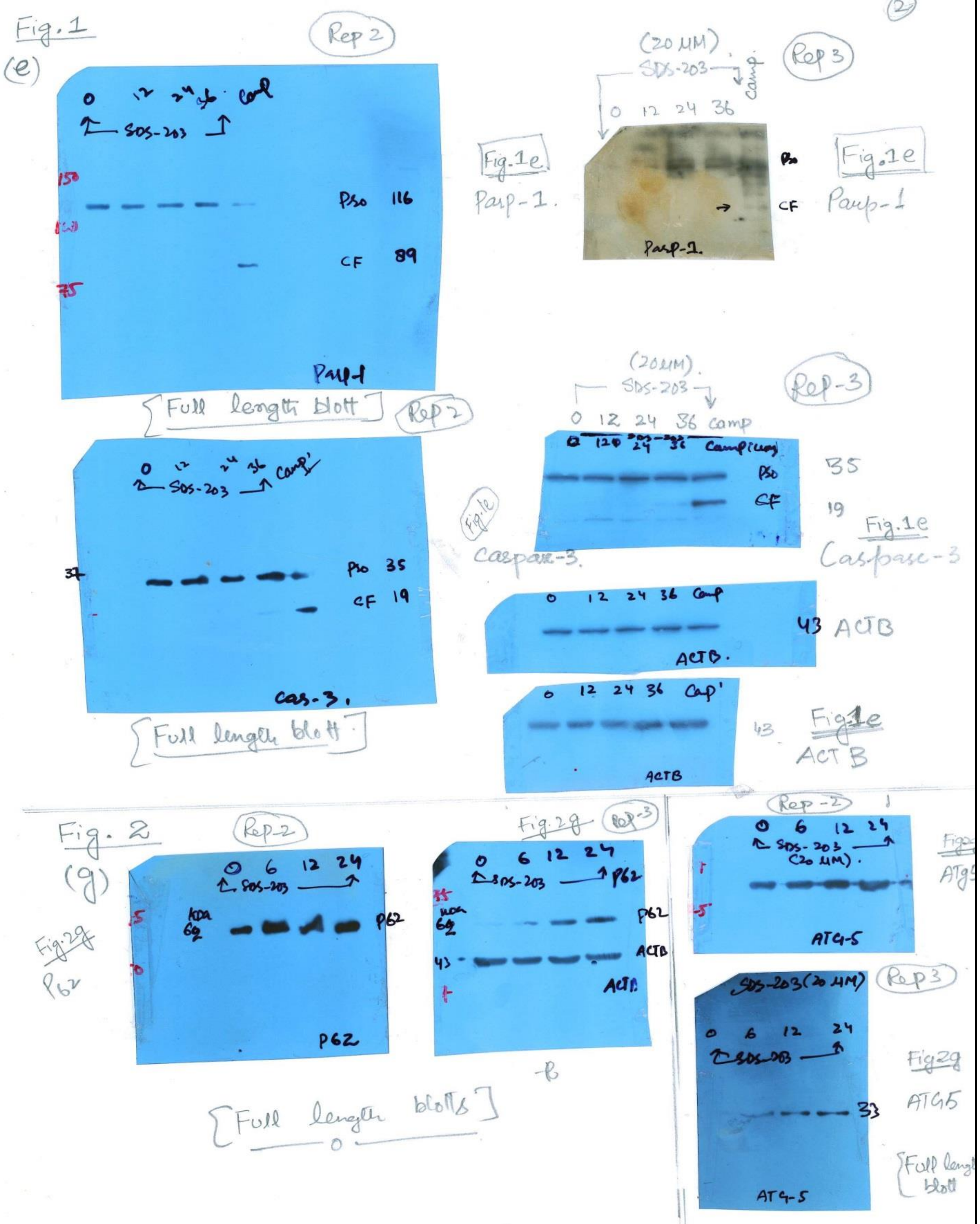

Fig. 1
(e)
Rep 2
0 12 24 36 Camp
SDS-203
150
75
Pso 116
CF 89
Parp-1
[Full length blott]
(20 µM)
SDS-203
camp
Rep 3
0 12 24 36
Fig. 1e
Parp-1
Pso
CF
Parp-1.
Rep 2
Pso 35
CF 19
Cas-3.
[Full length blott]
(20µM).
SDS-203
Rep-3
0 12 24 36 camp
Camp(1µg)
Fig. 1e
Caspase-3
Fig. 1e
Caspase-3
35
19
0 12 24 36 Camp
43 ACTB
ACTB.
0 12 24 36 Cap'
43
Fig 1e
ACT B
ACTB
Fig. 2
(g)
Rep-2
0 6 12 24
SDS-203
KDA
62
P62
Fig. 2g
P62
Fig. 2g
Rep-3
0 6 12 24
SDS-203
P62
ACTB
43
ACTB
Rep-2
0 6 12 24
SDS-203
(20 µM).
ATG-5
SDS-203 (20 µM)
Rep 3
0 6 12 24
SDS-203
33
Fig 2g
ATG5
ATG-5
[Full length blott]
[Full length blotts]

Fig. 2 (g)

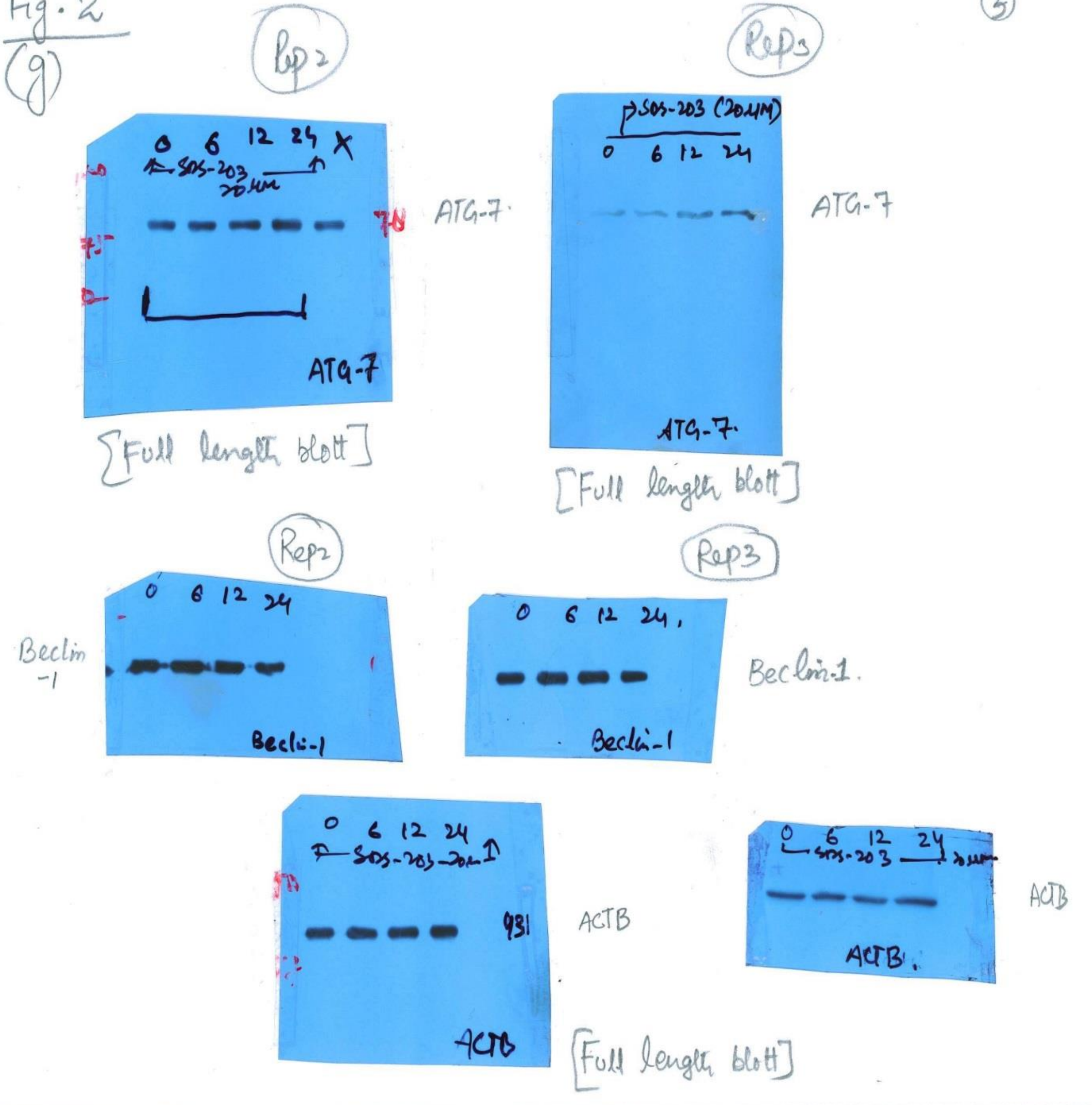

③
Rep2
Rep3
0 6 12 24 X
SDS-203 20µM
ATG-7
ATG-7
[Full length blott]
SDS-203 (20µM)
0 6 12 24
ATG-7
ATG-7
[Full length blott]
Rep2
Rep3
0 6 12 24
Beclin-1
Beclin-1
0 6 12 24
Beclin-1
Beclin-1
0 6 12 24
SDS-203 20µM
ACTB
ACTB
[Full length blott]
0 6 12 24
SDS-203 20µM
ACTB
ACTB

④

Fig-2

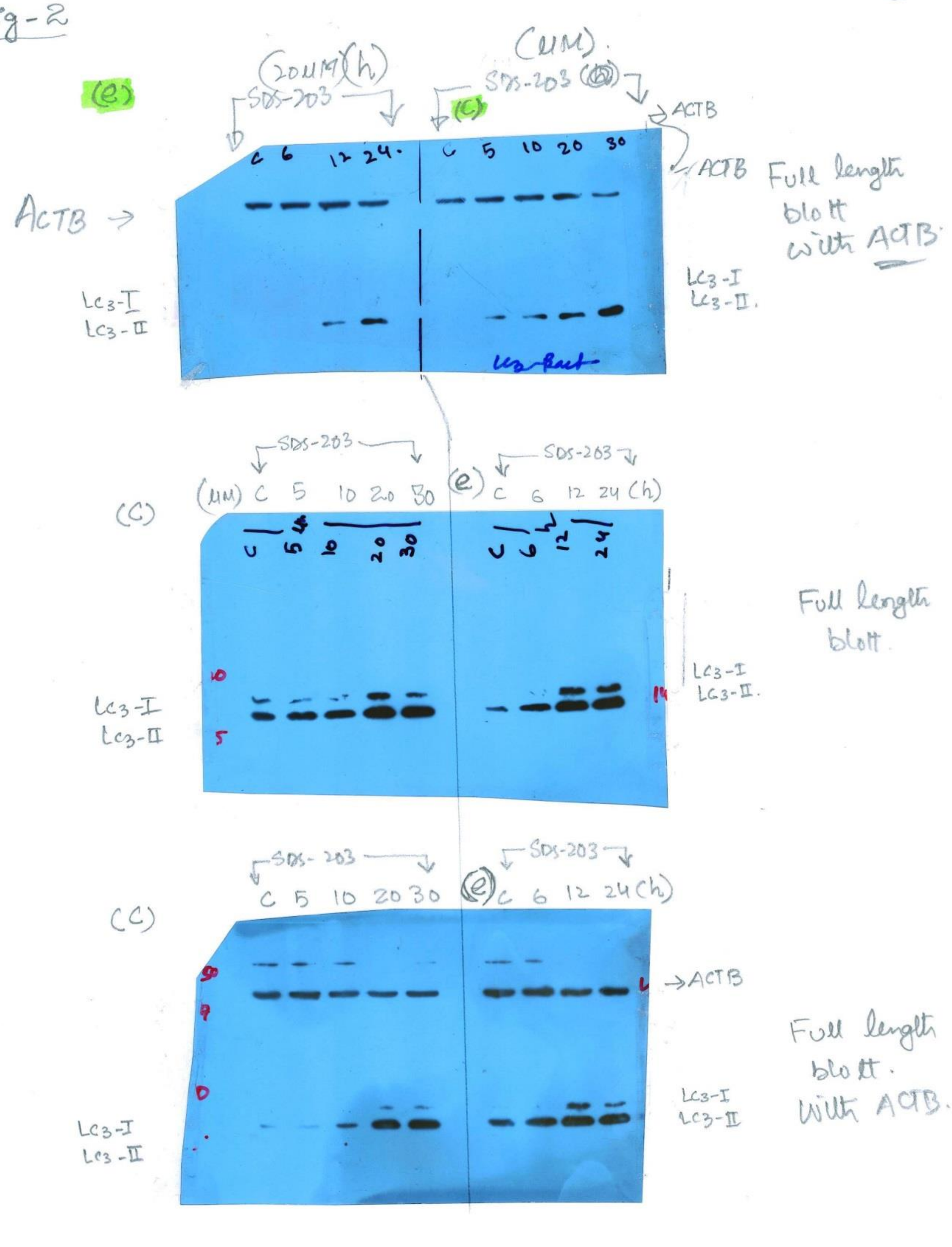
(e)
(20uM)(h)
SDS-203
(uM)
SDS-203
(c)
ACTB
c 6 12 24
c 5 10 20 30
ACTB
ACTB →
Full length blott with ACTB
Lc3-I
Lc3-II
Lc3-I
Lc3-II
SDS-203
(uM) C 5 10 20 30
(e) C 6 12 24 (h)
(C)
Full length blott.
Lc3-I
Lc3-II
Lc3-I
Lc3-II
SDS-203
C 5 10 20 30
(e) C 6 12 24 (h)
(C)
→ ACTB
Full length blott. With ACTB.
Lc3-I
Lc3-II
Lc3-I
Lc3-II

Main Figure

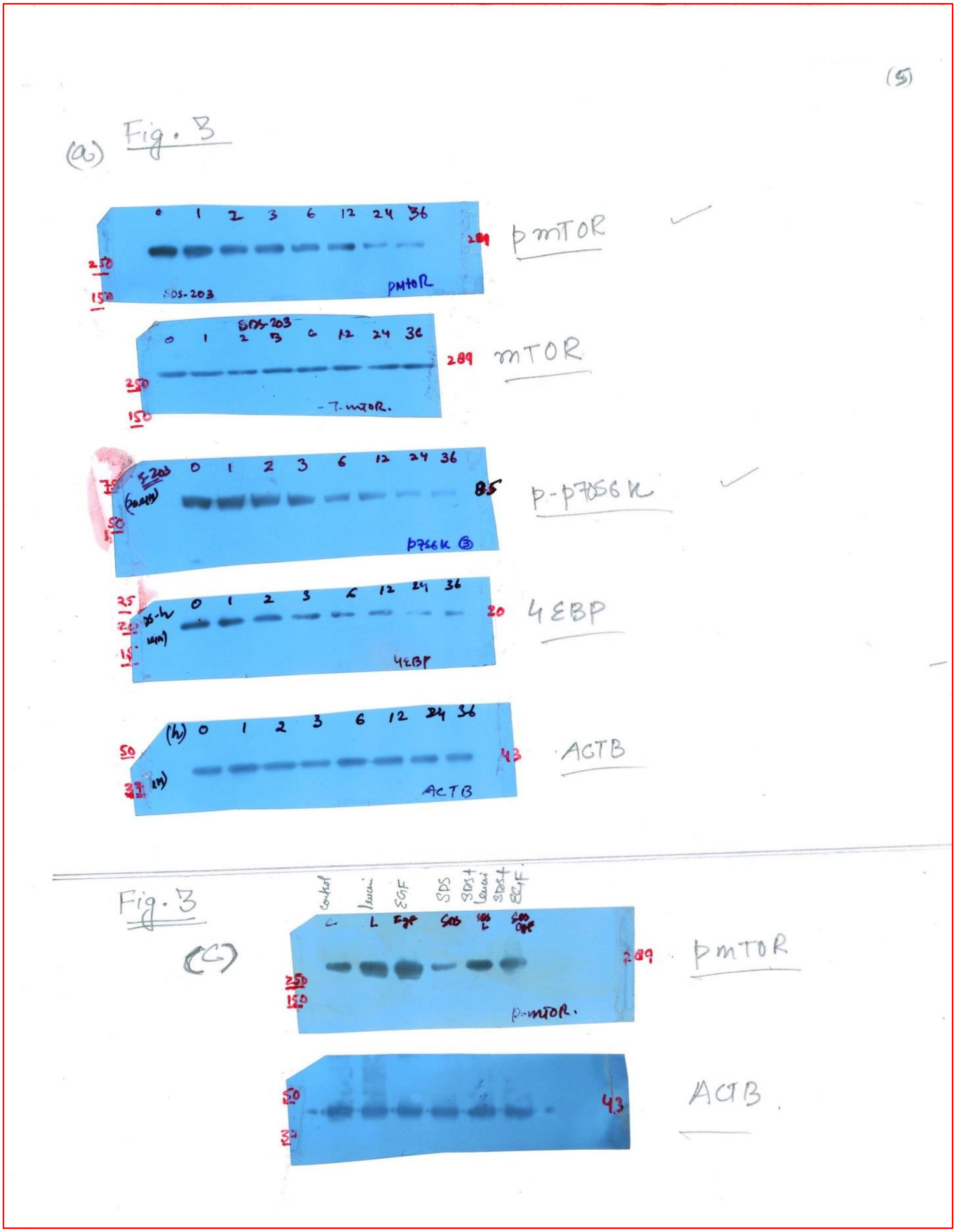
(5)
(a) Fig. 3
0 1 2 3 6 12 24 36
289
p mTOR
250
150
SDS-203
pmtoR
mTOR
250
150
p-p70S6K
85
75
50
4EBP
20
25
15
4EBP
ACTB
43
50
37
ACTB
Fig. 3
(C)
control
leucine
EGF
SDS
pmTOR
289
250
150
p-mTOR.
ACTB
50
43
37

Fig. 3

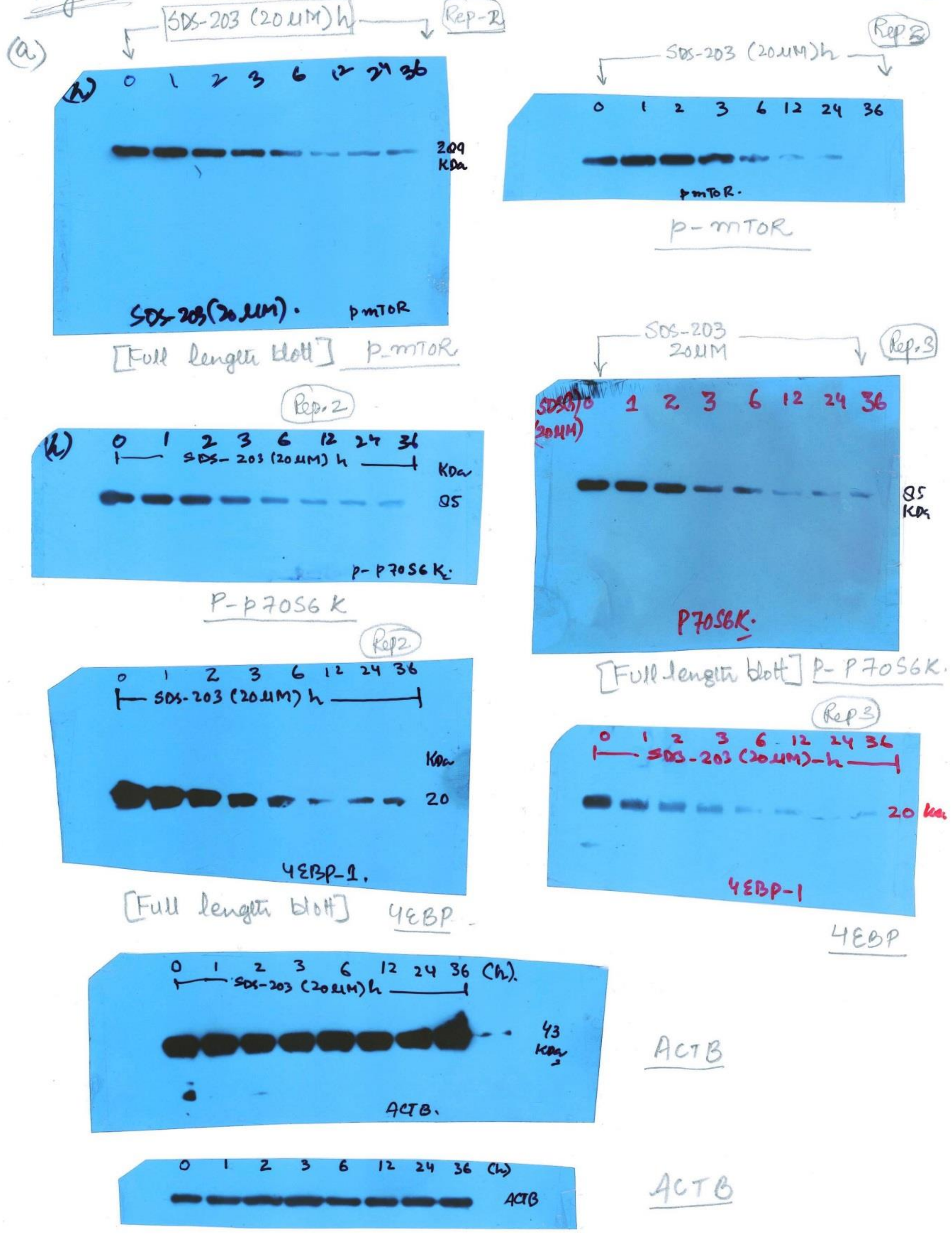
(a)
SDS-203 (20 uM) h
Rep-2
h 0 1 2 3 6 12 24 36
209 KDa
SDS-203 (20 uM). pmTOR
[Full length blott] P_mTOR
Rep 3
SDS-203 (20 uM) h
0 1 2 3 6 12 24 36
pmTOR.
p-mTOR
Rep. 2
h 0 1 2 3 6 12 24 36
SDS-203 (20 uM) h
KDa
85
p-P70S6K.
P-p70S6K
SDS-203 20 uM
Rep. 3
0 1 2 3 6 12 24 36
85 KDa
P70S6K.
[Full length blott] P-P70S6K.
Rep2.
0 1 2 3 6 12 24 36
SDS-203 (20 uM) h
KDa
20
4EBP-1.
[Full length blott] 4EBP.
Rep 3
0 1 2 3 6 12 24 36
SDS-203 (20 uM)-h
20 kDa
4EBP-1
4EBP
0 1 2 3 6 12 24 36 (h).
SDS-203 (20 uM) h
43 KDa
ACTB.
ACTB
0 1 2 3 6 12 24 36 (h)
ACTB
ACTB

(Fig. 3)

(C)

Rep-2

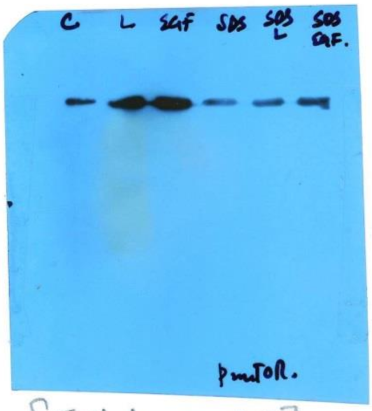

289 kDa

p mTOR

[Full length blott]

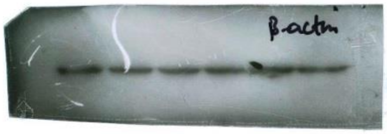

43KDa ACTB'

Rep-3

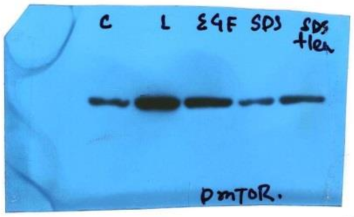

289 kDa

p mTOR

[Without EGF + SDS Sample]

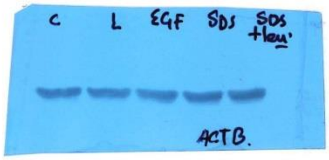

43kDa

ACTB

Fig 3(a)

SDS-203 (20 μM)

Rep-2

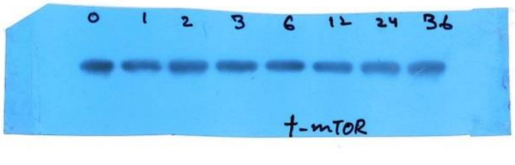

289

mTOR

Rep 3

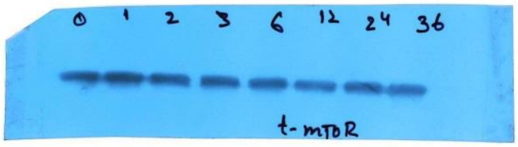

289

mTOR

Main Figure

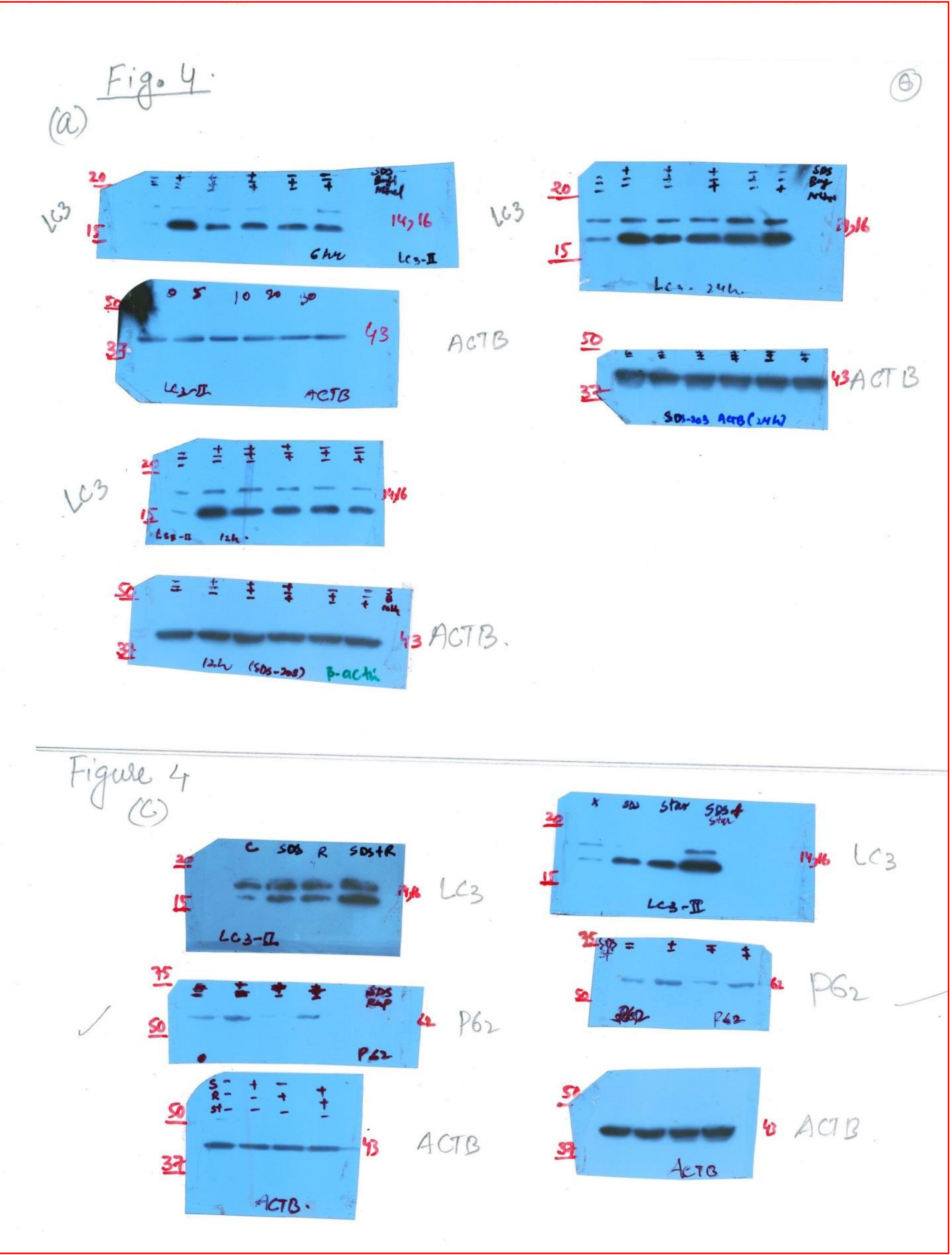
Fig. 4.
(a)
LC3
ACTB
ACTB
LC3
ACTB.
12h (SDS-203) β-actin
LC3
ACTB
Figure 4
(C)
C SDS R SDS+R
LC3
LC3-II
P62
ACTB
ACTB.
LC3
LC3-II
P62
ACTB

Fig - 4. (a)

(a) Rep 2 — 24h

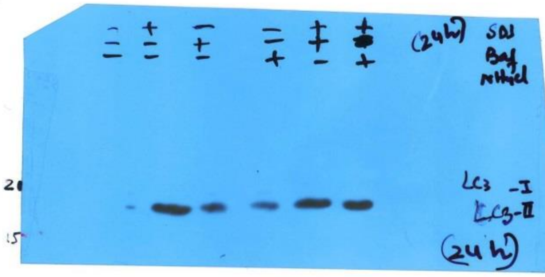

LC3-I
LC3-II

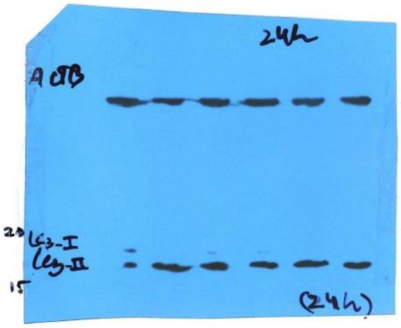

ACTB

LC3-I
LC3-II

[Full length blott] [Full length blott] with ACTB.

(a)

Rep 2 — 12h

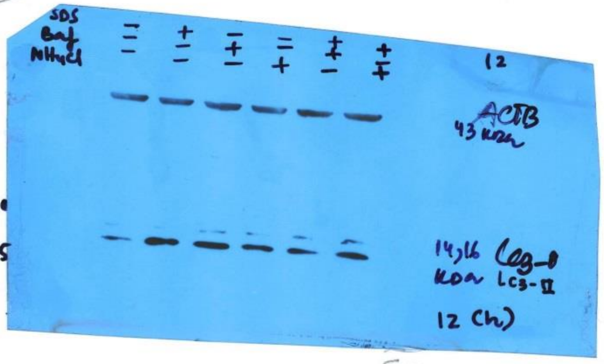

ACTB (43KDa)

LC3-I
LC3-II

[Full length blott] with ACTB

(a) Rep2 — 6h

ACTB →

LC3-I >
LC3-II :

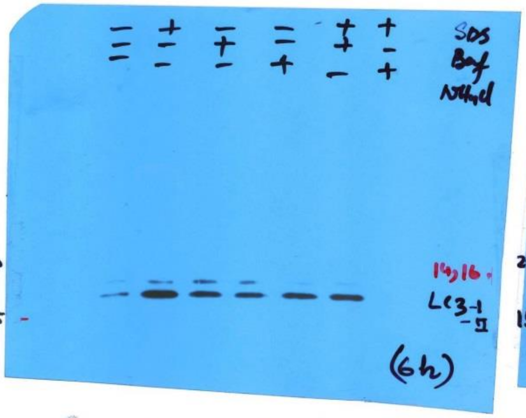

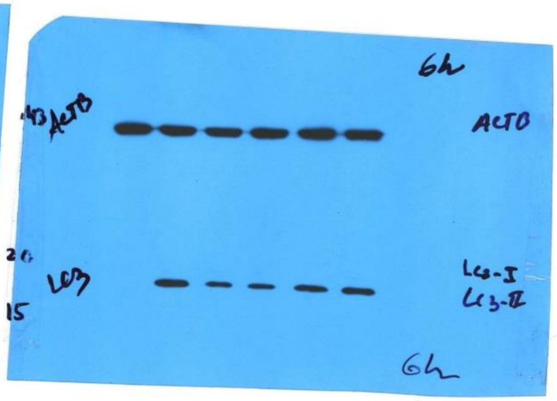

LC3-I
LC3-II

[Full length blott] [Full length blott] with ACTB

Fig. 4.

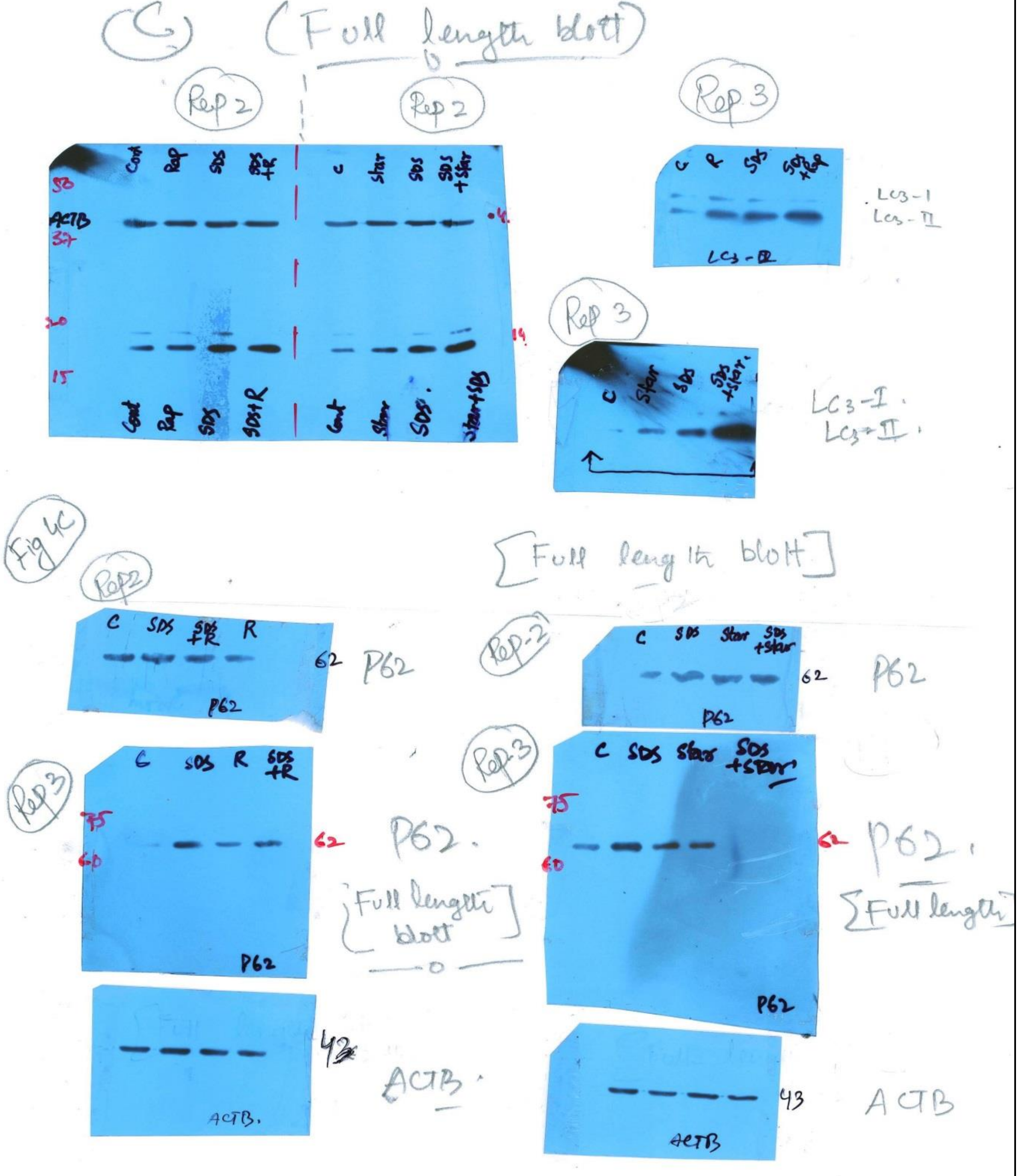
(C) (Full length blott)
Rep 2
Rep 2
Rep 3
Cont Rep SDS SDS+R
C Starv SDS SDS+Starv
ACTB
50
37
20
15
LC3-I
LC3-II
Rep 3
C Starv SDS SDS+Starv
LC3-I
LC3-II
Fig 4C
[Full length blott]
Rep2
C SDS SDS+R R
62
P62
Rep-2
C SDS Starv SDS+Starv
62
P62
Rep3
C SDS R SDS+R
75
62
60
P62.
[Full length blott]
Rep3
C SDS Starv SDS+Starv
75
62
60
P62.
[Full length]
42
ACTB.
43
ACTB

Main Figure

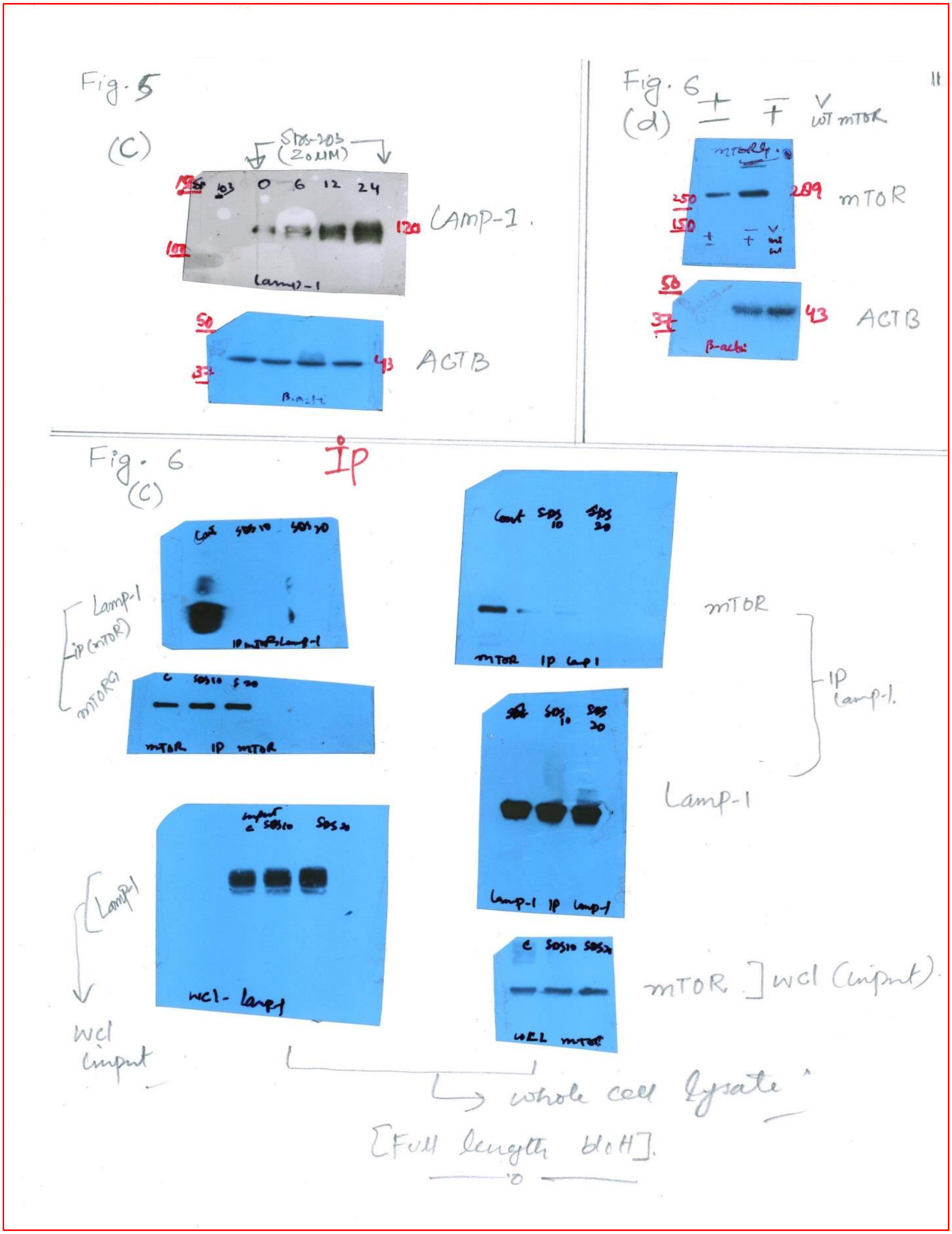

Fig. 5
(C)
Lamp-1
ACTB
Fig. 6
(d)
mTOR
ACTB
Fig. 6
(C)
IP
mTOR
IP Lamp-1
Lamp-1
mTOR] WCl (input)
WCl input
whole cell lysate
[Full length blot]

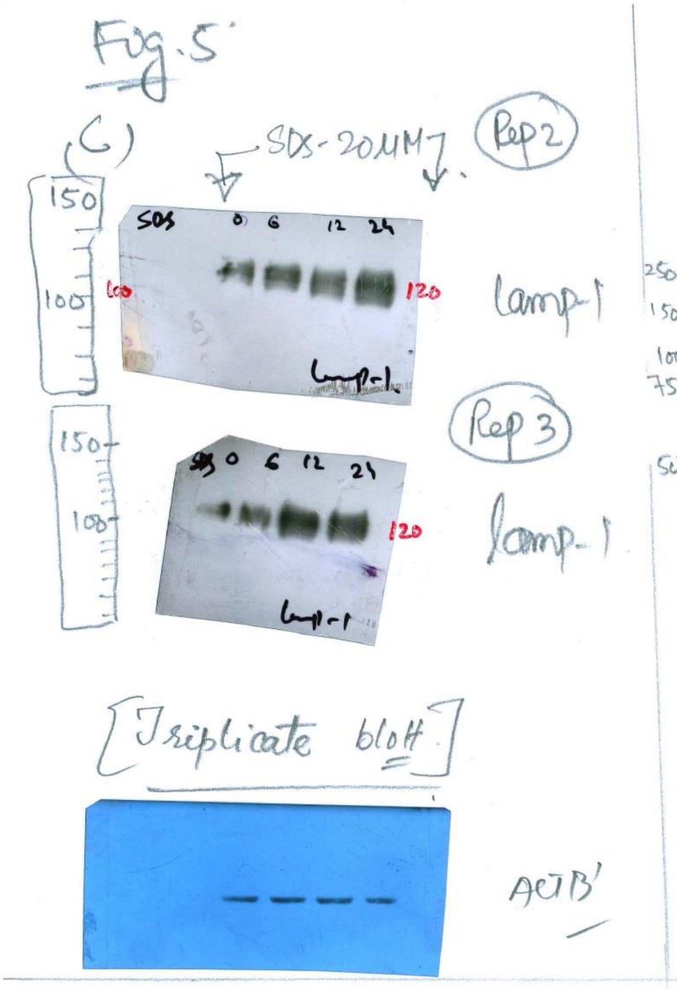
Fig. 5
(C)
SDS-20MM
Rep 2
SDS 0 6 12 24
150
100
100
120
lamp-1
Lamp-1
Rep 3
SDS 0 6 12 24
150
100
120
lamp-1
Lamp-1
[Triplicate blott]
ACTB'

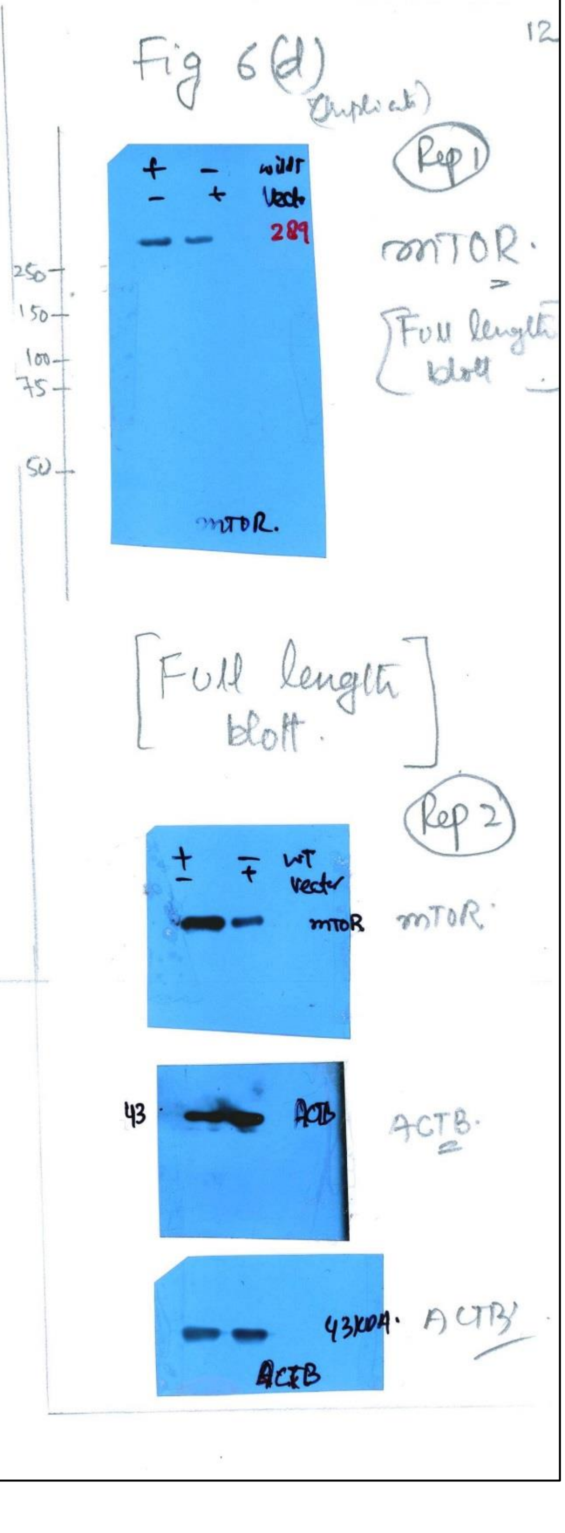
Fig 6(d)
(Duplicate)
12
Rep 1
+ − wildT
− + Vector
289
mTOR.
[Full length blott]
250
150
100
75
50
mTOR.
[Full length blott.]
Rep 2
+ − WT
− + vector
mTOR
mTOR.
43
ACTB
ACTB.
43KDA.
ACTB'
ACTB

Fig 6 (C) [IP].

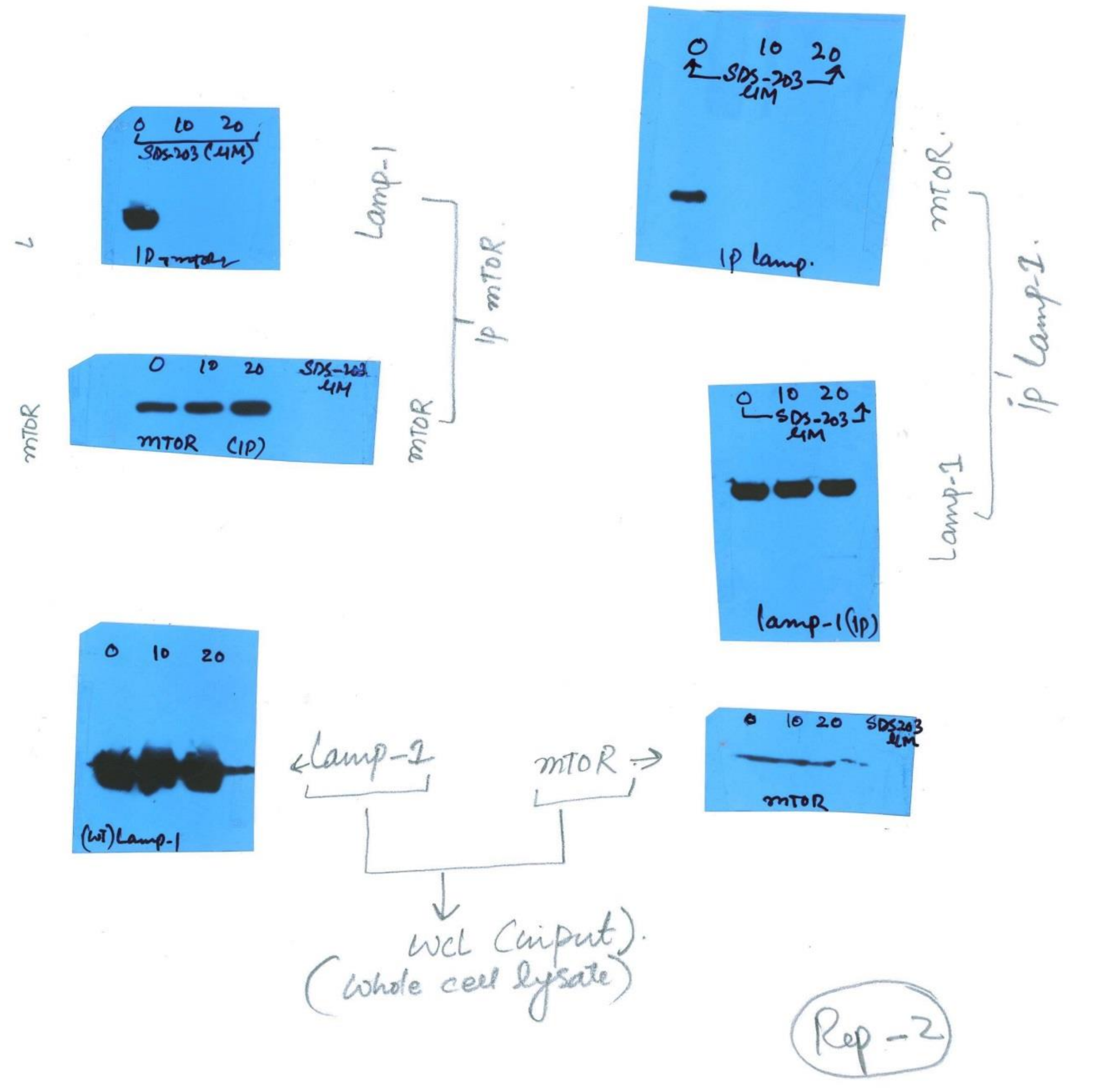
0 10 20
SDS-203 (µM)
IP mTOR
Lamp-1
IP mTOR
0 10 20 SDS-203 µM
mTOR (IP)
mTOR
mTOR
0 10 20
SDS-203 µM
IP lamp.
mTOR.
IP Lamp-1.
0 10 20
SDS-203 µM
lamp-1 (IP)
Lamp-1
0 10 20
(WT) Lamp-1
Lamp-1
mTOR
0 10 20 SDS203 µM
mTOR
WCL (input).
(whole cell lysate)
Rep-2

Main Figure

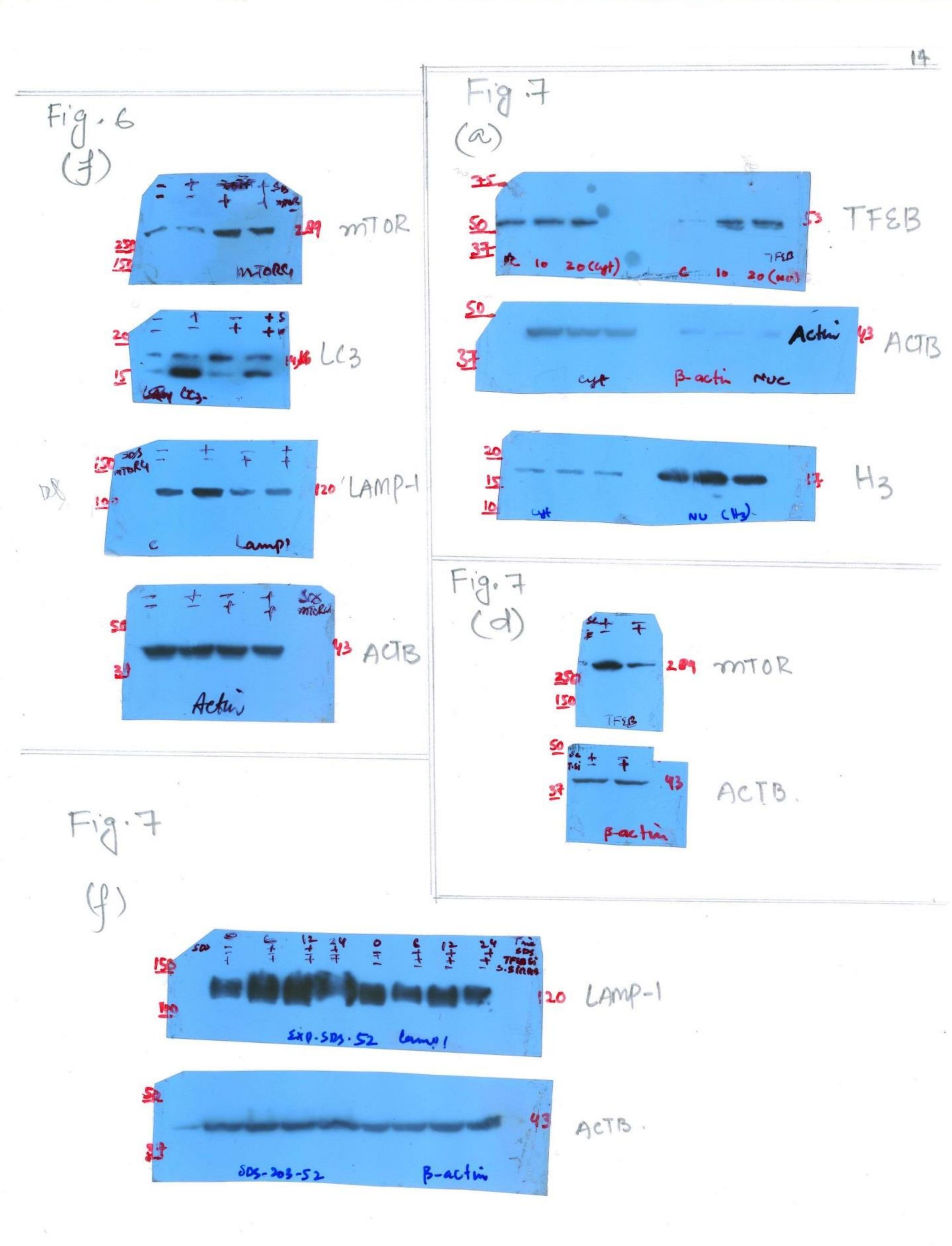

14
Fig. 6
(f)
mTOR
LC3
LAMP-1
ACTB
Fig. 7
(a)
TFEB
ACTB
H3
Fig. 7
(d)
mTOR
ACTB.
Fig. 7
(f)
LAMP-1
ACTB.

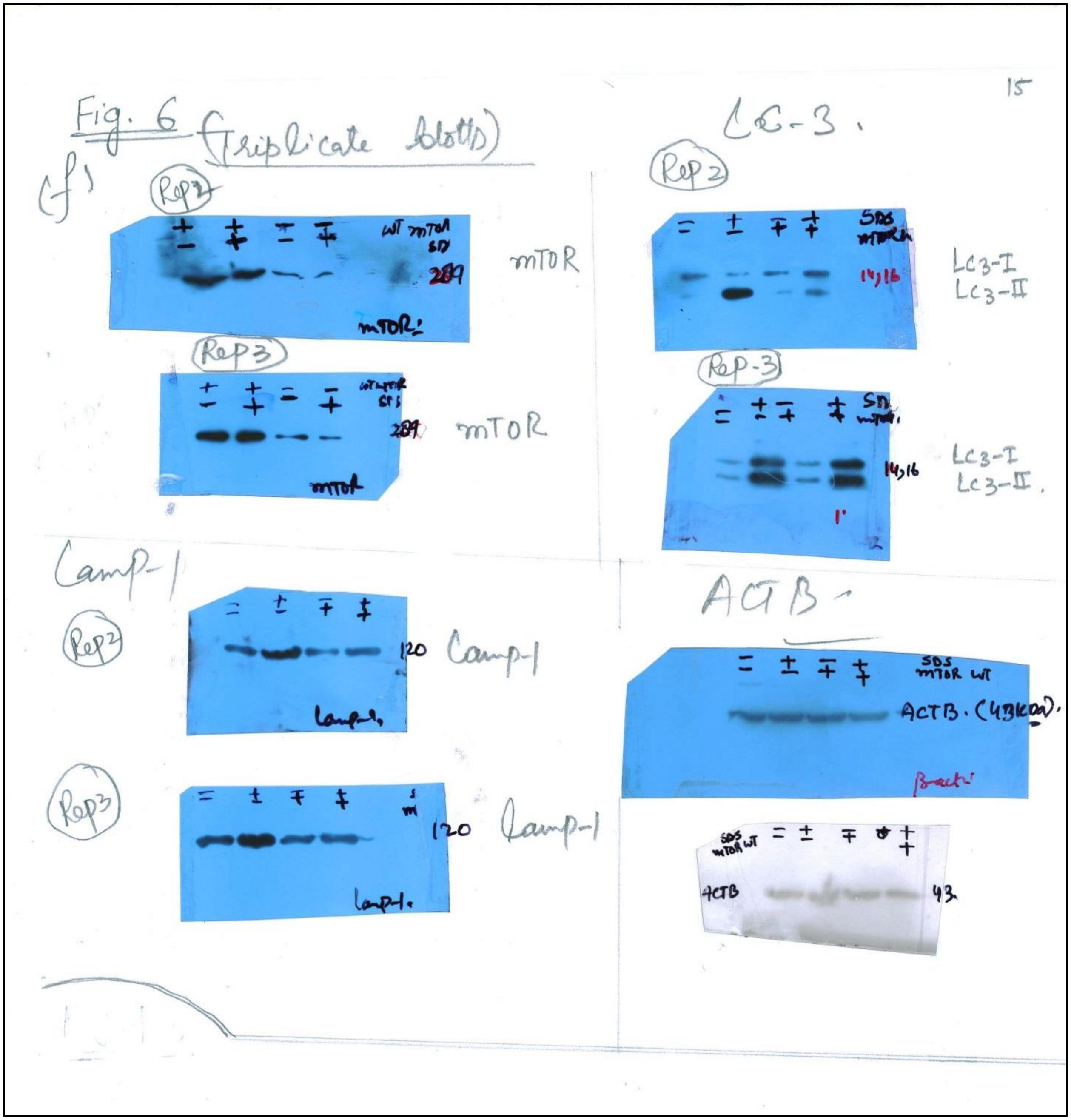
15
Fig. 6 (Triplicate blots)
(f)
Rep 2
287
mTOR
Rep 3
287
mTOR
LC-3.
Rep 2
14,16
LC3-I
LC3-II
Rep-3
14,16
LC3-I
LC3-II.
Lamp-1
Rep2
120 Lamp-1
Rep3
120 Lamp-1
ACTB
ACTB. (43kDa).
ACTB
43.

Fig. 7a

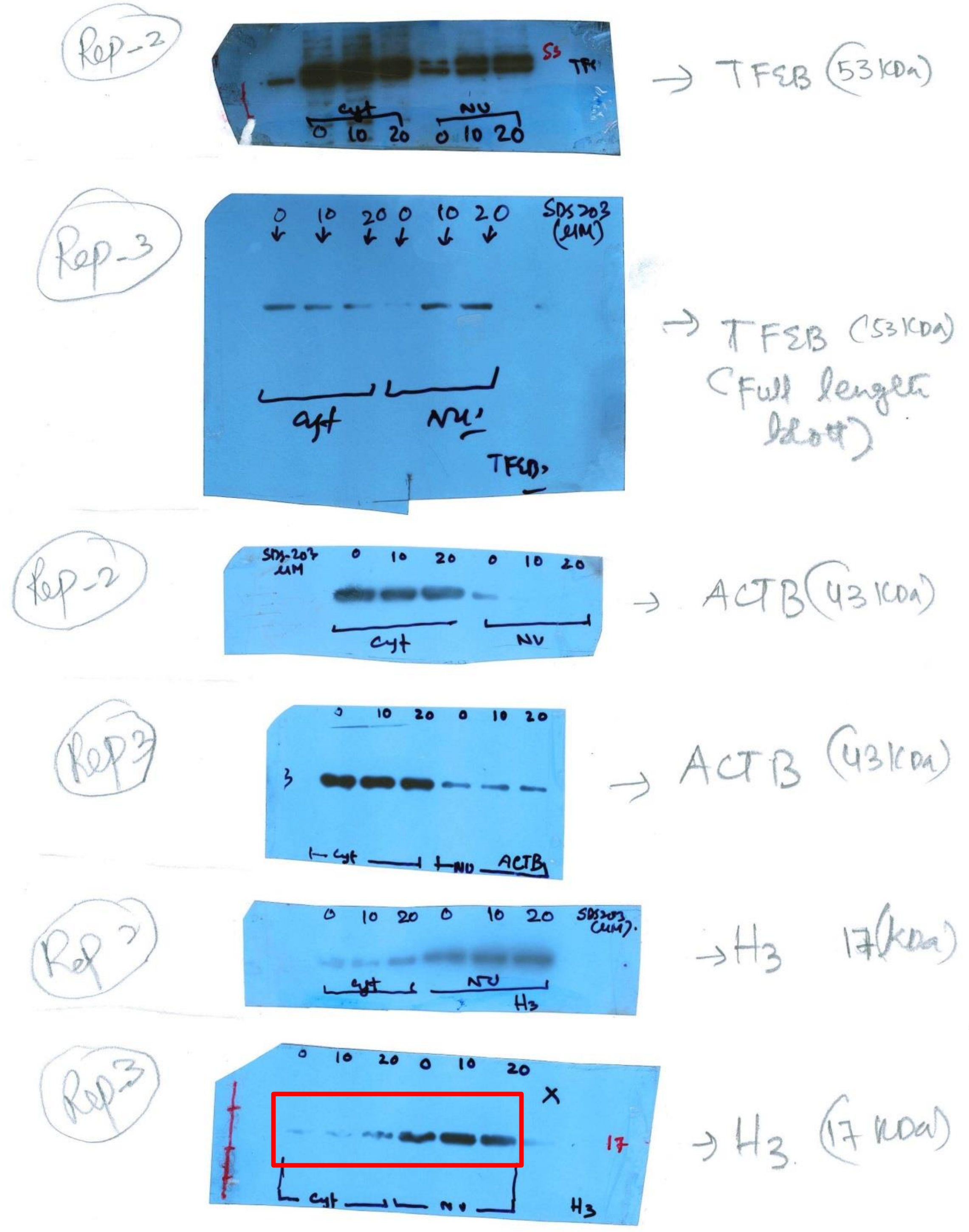

Rep_2
cyt
NU
0 10 20 0 10 20
→ TFEB (53 KDa)
Rep_3
0 10 20 0 10 20
SDS 203 (uM)
cyt
NU
→ TFEB (53 KDa) (Full length blott)
Rep_2
SDS-203 uM
0 10 20 0 10 20
cyt
NU
→ ACTB (43 KDa)
Rep 3
0 10 20 0 10 20
cyt
NU
ACTB
→ ACTB (43 KDa)
Rep 2
0 10 20 0 10 20
SDS 203 (uM).
cyt
NU
H3
→ H3 17 (KDa)
Rep 3
0 10 20 0 10 20
cyt
NU
H3
17
→ H3 (17 KDa)

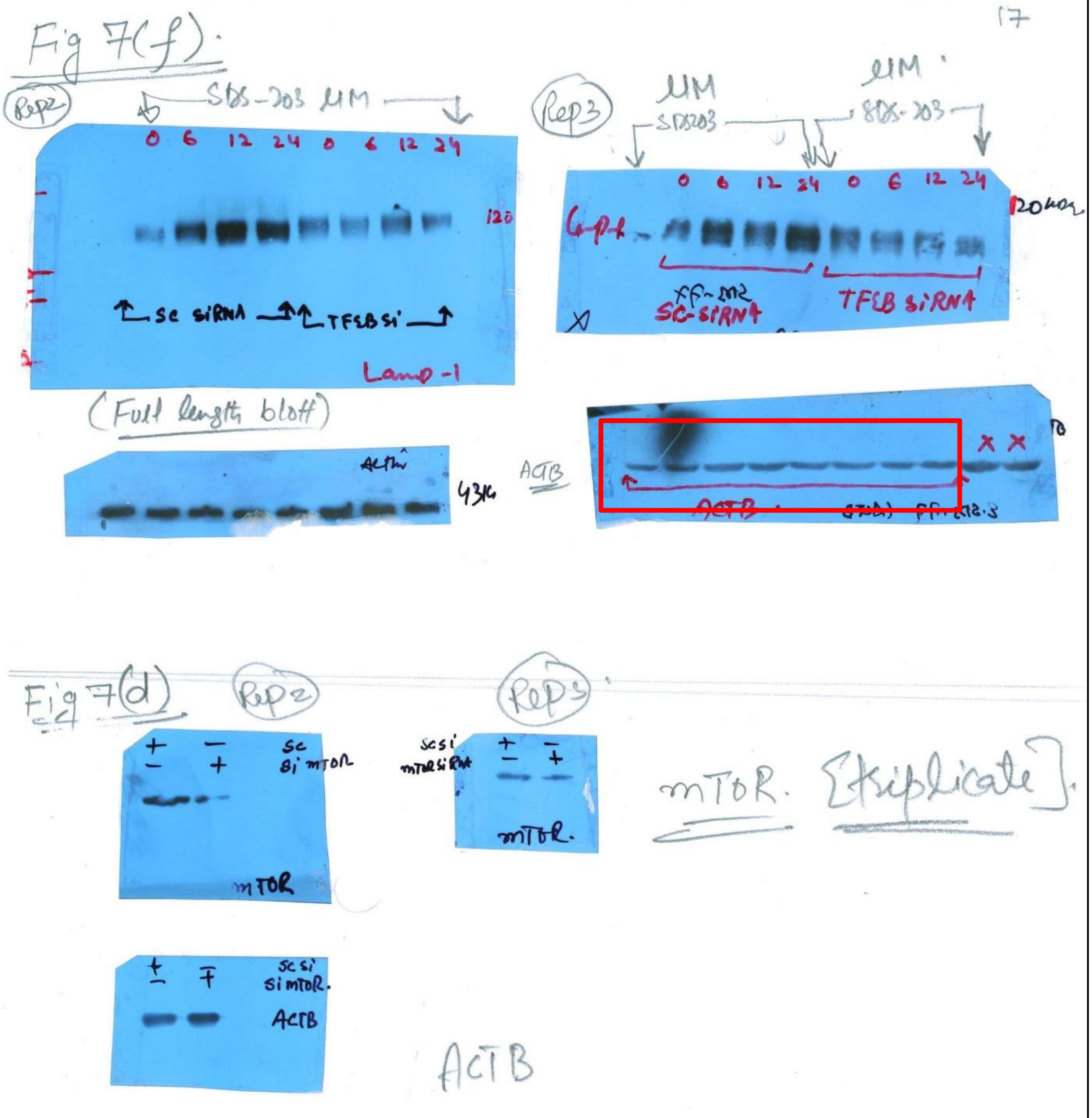
17
Fig 7(f).
Rep2
SDS-203 μM
0 6 12 24 0 6 12 24
120
Sc siRNA
TFEB si
Lamp-1
(Full length blott)
Actin
43k
Rep3
μM
SDS203
μM
SDS-203
0 6 12 24 0 6 12 24
120kDa
SC-SiRNA
TFEB SiRNA
ACTB
Fig 7(d)
Rep2
Rep3
Sc
Si mTOR
mTOR
Scsi
mTOR SiRNA
mTOR.
mTOR. [triplicate]
Sc si
si mTOR.
ACTB
ACTB

Main Figure

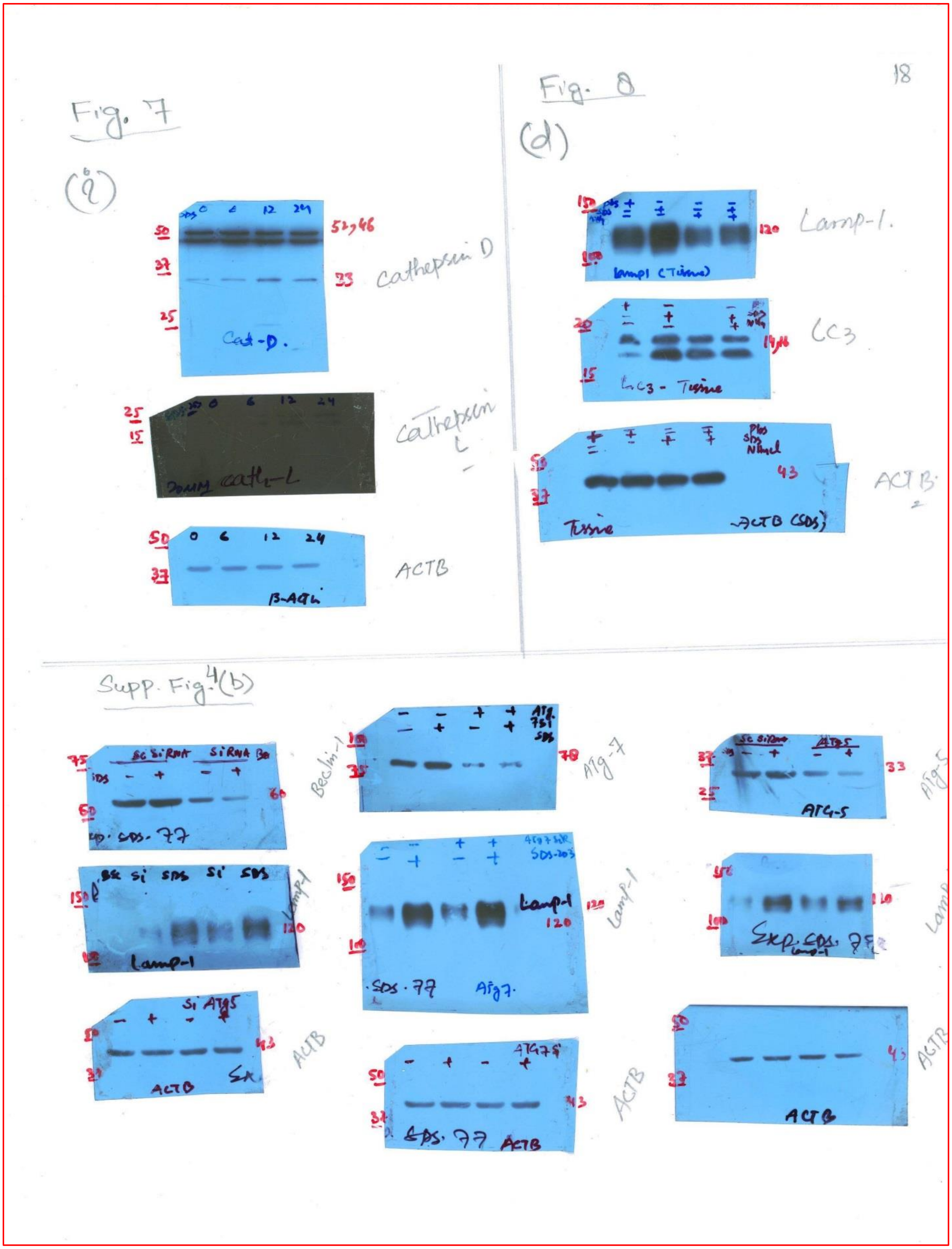
Fig. 7
Cathepsin D
Cat-D.
Cathepsin L
ACTB
Fig. 8
(d)
18
Lamp-1.
LC3
LC3 - Tissue
ACTB
Tissue
ACTB (SDS)
Supp. Fig. 4(b)
Beclin-1
Atg-7
Atg-5
ATG-5
Lamp-1
ACTB
SDS. 77
Atg7.
ACTB

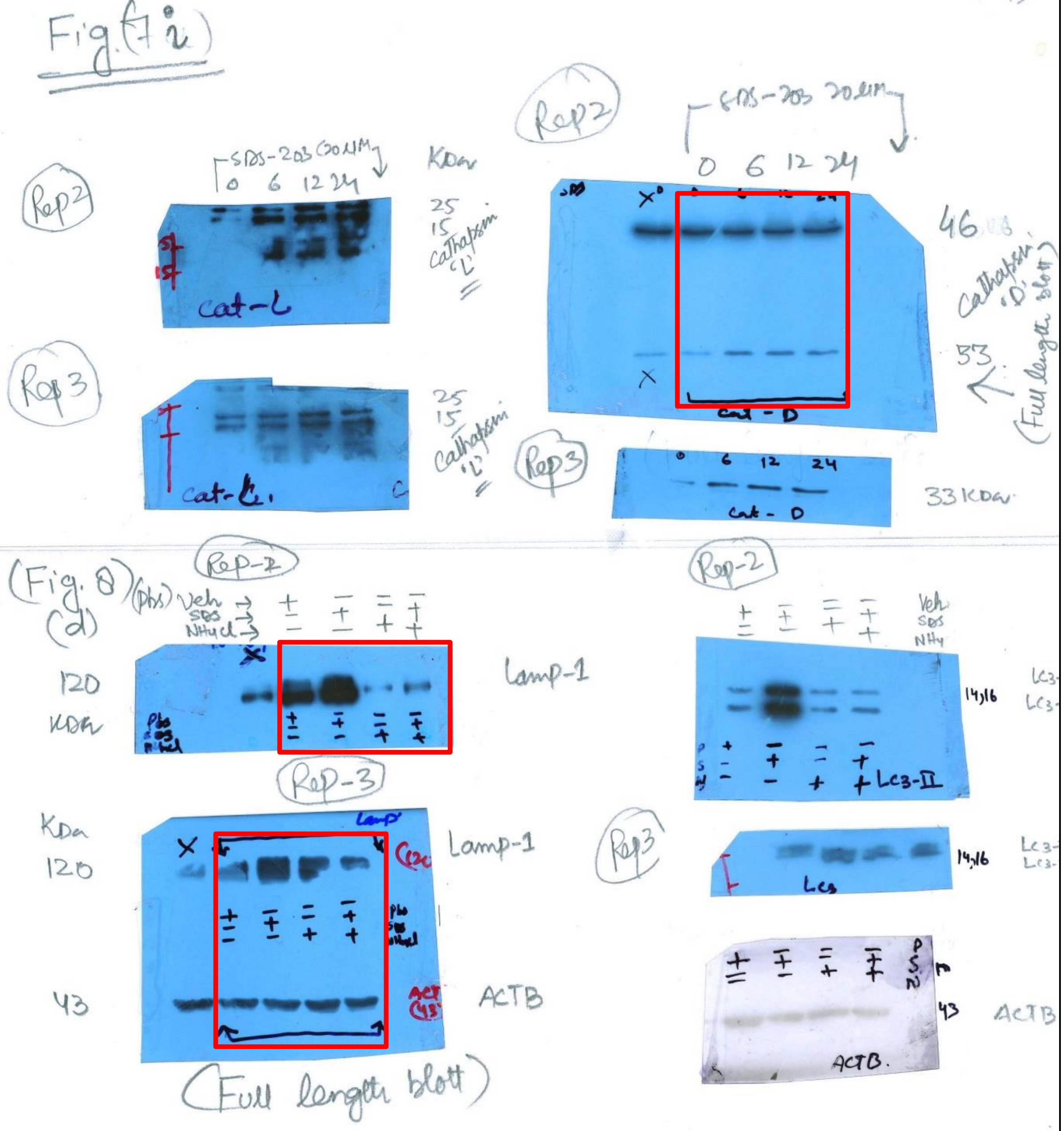
Fig. (7 i)
Rep2
SDS-203 (20 μM)
0 6 12 24
KDa
25
15
Cathapsin 'L'
cat-L
Rep3
cat-L
SDS-203 20 μM
46
Cathapsin 'D'
(Full length blott)
33
cat-D
Rep3
0 6 12 24
33 KDa
(Fig. 8) (d)
Rep-2
(pbs) Veh →
SDS →
NH4Cl →
120
KDa
Lamp-1
Rep-3
KDa
120
Lamp-1
ACTB
43
(Full length blott)
Veh
SDS
NH4
14,16
LC3-II
Rep3
Lc3
43
ACTB

Supp. Fig. 4(b) (Triplicate blots) 20

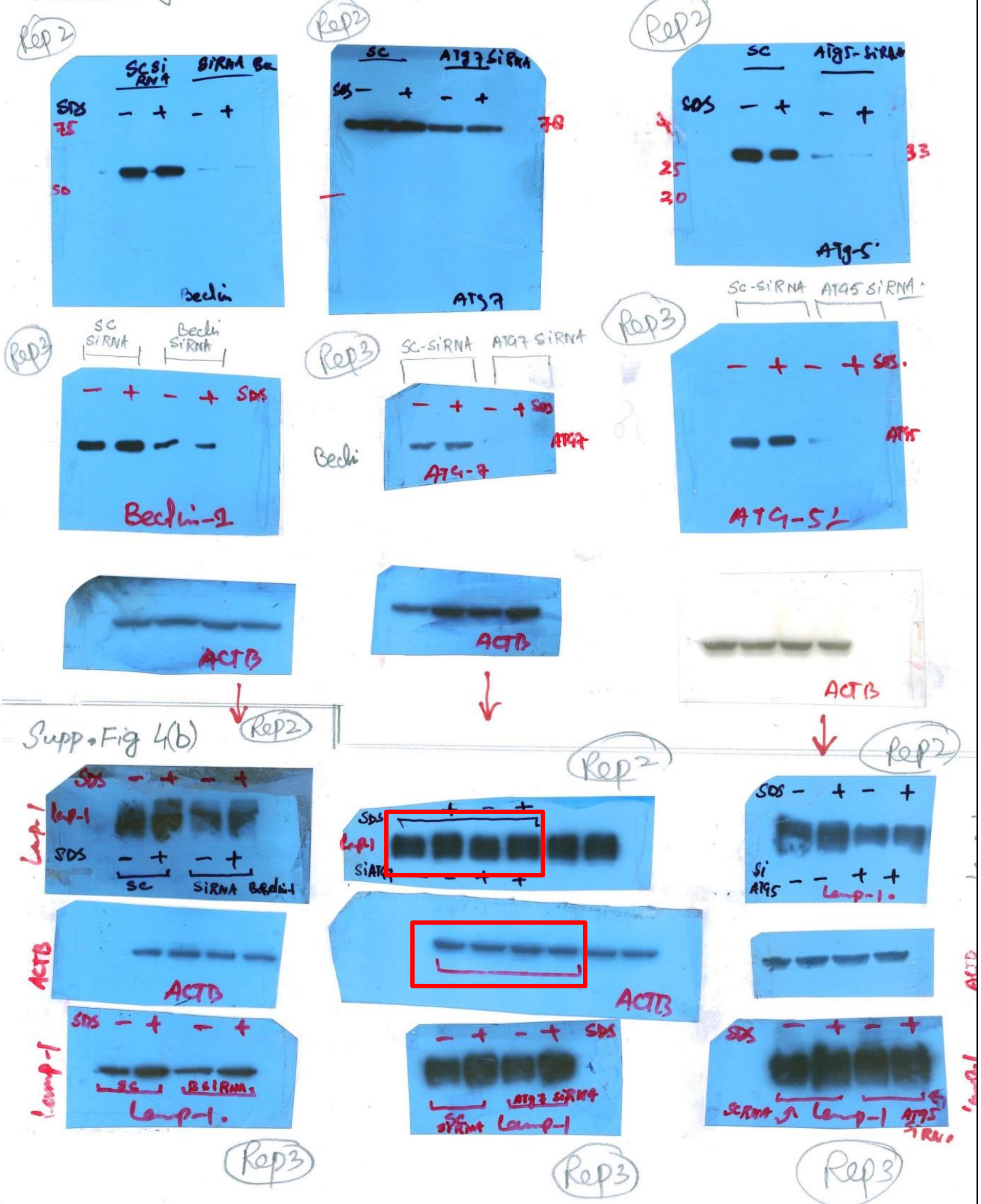

Supplement: Supplementary file 1 — Supplementary Figures. [file 41598_2022_7955_MOESM1_ESM.pdf]
